# From Metabolically Healthy to Unhealthy Obesity Through Low-Grade Inflammation

**DOI:** 10.3390/biomedicines14051161

**Published:** 2026-05-20

**Authors:** Anastasia Voznesenskaya, Alyona Sorokina, Marina Shestakova, Ekaterina Shestakova, Ildar Minniakhmetov, Anna Ivanova, Sergey Rumyantsev, Natalia Mokrysheva, Vladimir Chekhonin, Marina Loguinova

**Affiliations:** Endocrinology Research Centre, 117292 Moscow, Russia

**Keywords:** morbid obesity, type 2 diabetes mellitus (T2DM), metaflammation, bariatric surgery, obesogenic memory, CRP, IL-6, IL-17A, senescent immune cells

## Abstract

Of the many clinical phenotypes of obesity, the most prevalent are metabolically “healthy” (MHO) and metabolically “unhealthy” (MUO) obesities, the latter being associated with a range of comorbidities, including type 2 diabetes mellitus (T2DM). The underlying causes of different obesity phenotypes and the mechanisms of conversion of one phenotype into another have yet to be fully elucidated. However, increasing evidence suggests the key role of low-grade metabolic inflammation (metaflammation) in the pathogenesis of obesity and metabolic dysfunction. The review presents a comprehensive description of changes in immune cell populations and pro-inflammatory mediators, as well as a detailed comparative mapping of the adipose tissue immune landscape during MHO/MUO transition. Based upon a conceptual model for the intensification of metaflammation during MHO progression and conversion to MUO, a pattern of dynamical changes that accompany MHO/MUO transition is described. Though many parameters demonstrate significant differences in multiple cross-sectional and some longitudinal studies, only a few of them (CRP, IL-6, IL-17A, absolute counts of leukocytes and neutrophils) meet the criteria of a validated biomarker in clinical setting. A lack of standardization in MHO definition and heterogeneity in the severity of MUO make the search for predictive biomarkers a challenge. The review also discusses the mechanisms underlying metabolic memory and the incomplete reversibility of metabolic disturbances after bariatric surgery.

## 1. Introduction

In recent decades, the global prevalence of obesity has been steadily increasing, contributing to diminished quality of life, reduced life expectancy, and increased disability due to its comorbidities—type 2 diabetes mellitus (T2DM), cardiovascular disease, cancer, etc. At the same time, it has been demonstrated that not all obese individuals develop metabolic and cardiovascular disorders. This has resulted in the specification of two obesity phenotypes: metabolically “healthy” obesity (MHO) and metabolically “unhealthy” obesity (MUO). Some researchers have additionally identified specific obesity categories, such as “metabolically unhealthy non-obese” (MUNO) individuals, who have normal body mass index (BMI) but excessive visceral adiposity, insulin resistance (IR), and impaired glucose metabolism, as well as “sarcopenic obesity”, characterized predominantly by muscle mass loss [1]. This review concerns two fundamentally distinct phenotypes of obesity: MHO and MUO.

The concept of obesities of different severity arose a long time ago and has been reconsidered from different perspectives. In early 2025, the International Commission of The Lancet Diabetes & Endocrinology proposed distinguishing “preclinical” obesity, not accompanied by organ dysfunction, from “clinical” obesity, complicated by a spectrum of associated diseases, including metabolic disorders [2]. This raises several questions: why do some individuals with abnormal BMI not suffer from metabolic disorders, while others develop T2DM over time? What factors contribute to the conversion from “metabolic health” to its impairment? Is the progression from MHO to MUO inevitable and merely a matter of time [3,4,5]? What metabolic, immunological, and hormonal changes accompany this transition? Is it possible to revert to a “metabolically healthy” state after weight loss and T2DM remission following bariatric surgery? Does this depend on the patient’s current metabolic, immunological, and hormonal signature? Is it influenced by the duration of obesity?

It is estimated that MHO progresses to MUO in approximately 60% of cases over a 10-year period [6]. Ler et al. observed a transition from MHO to MUO in 56% of cases in a 27-year follow-up [5]. The authors also reported conversion of MUO to MHO in 54% of cases. However, the accompanying clinical and immunological changes during such transitions are not discussed in the study. At the same time, several comprehensive reviews have compared MHO and MUO, highlighting factors contributing to obesity phenotype and shift of MHO towards MUO. The main reasons for the change in the obesity phenotype include lifestyle, genetic factors [7], the predominance of visceral adipose tissue (VAT) [8], structural alterations and inflammation in VAT [9,10,11] accompanied by increased pro-inflammatory factors and inflammatory circulating cells [12,13,14]. Blüher, in his review, analyzed several biological factors contributing to MUO and its transition from MHO [15]. Other reviews have focused on surgical and therapeutic approaches to MUO treatment [16,17] The review by Duque et al. is notable for the analysis of inflammatory biomarker patterns (neutrophil-to-lymphocyte ratio, levels of soluble urokinase plasminogen activator receptor (suPAR), or gut and adipose tissue (AT) hormones) for the prevention of cardiovascular disease in patients with MHO [18].

In the present review, we define MHO and MUO phenotypes and analyze the role of various factors in the development of these obesity types. Given that obesity is accompanied by low-grade metabolic inflammation (or metaflammation), we examine MHO and MUO from its perspective, comparing both types of obesity using multiple inflammation-associated parameters. The review presents a uniquely comprehensive and detailed description of changes in immune cell populations (neutrophils, eosinophils, mast cells, macrophages, T and B lymphocyte subpopulations, NK cells, ILCs) during the transition from MHO to MUO. Upon analyzing a number of clinical, biochemical, and hormonal parameters, and comparing the number, frequency, and functional activity of various immune cell subsets in the circulation and AT, we reveal that many parameters demonstrate significant differences between MHO and MUO in multiple cross-sectional studies. However, only a few inflammatory mediators and circulating cells meet the criteria of a validated biomarker. The review also contributes to the discourse on the incomplete reversibility of metaflammation following bariatric interventions.

## 2. Differences in Metabolic Status and the Role of General Factors Including Sex, Duration of Obesity, Fat Distribution, and Lifestyle in the Formation of Obesity Phenotypes

A recent meta-analysis of 12 cohort and 7 interventional studies demonstrated that MHO makes up 35% of individuals with obesity, with significant regional variation [19]. Notably, the prevalence of MHO depends heavily on the criteria used to identify metabolic healthy obese individuals. These criteria remain largely lacking in standardization. In some studies, the primary criterion was the absence of T2DM or specific fasting serum glucose thresholds [20]. The majority of research has assessed insulin resistance through measures such as homeostasis model assessment of insulin resistance (HOMA-IR), the hyperinsulinemic–euglycemic clamp, oral glucose tolerance test (OGTT), fasting serum insulin levels, or insulin suppression tests [3]. In certain investigations, insulin sensitivity alone served as the criteria for metabolic normality [8], whereas other studies selected favorable levels of HDL cholesterol, triglycerides, blood pressure, serum uric acid, C-reactive protein, plasma fibrinogen, and white blood cell count, among other factors [21]. Some research has also defined metabolic health based on the presence or absence of metabolic syndrome [22]. Additionally, the cutoff values employed across studies displayed considerable variability—some used thresholds above the highest quartile of HOMA-IR, others used tertiles, or the 90th percentile, while some studies adopted alternative cutoff points. Most of these investigations were cross-sectional and consistently reported that MHO individuals exhibit a favorable distribution of body fat, more advantageous hormonal and lipid profiles, and lower levels of proinflammatory cytokines and adipokines [3].

A recent analysis from a database containing the data of 231,399 patients with a recorded BMI of ≥35 kg/m^2^, suggested that men are more prone to conversion from MHO to MUO, HR = 1.43, CI 1.41–1.45, *p* = < 0.01 [23]. At the same time data from the Northwest Adelaide Health Study, which included 3743 women (51%) and men, showed that reverse transition from MUO to MHO occurred without significant gender differences in 16% of the participants in recall visits of up to 10 years [4]. There is a tendency for MUO conversion in older people, supporting the role of obesity duration in metabolic complications [24]. The higher prevalence of obesity-related comorbidities in postmenopausal compared with premenopausal women [25] suggests that declining sex hormone levels may also play a significant role. Sufficient physical activity can reduce the risk of T2DM and cardiovascular diseases [9,26]. In a large BMI-stratified cohort, Stefan N and colleagues [27] associated excessive hepatic and abdominal fat accumulation with MUO, whereas, in subcutaneous and predominantly lower extremities, fat accumulation was found to be associated with MHO. The protective role of subcutaneous fat has also been reported by other authors [28].

It is commonly hypothesized that excess caloric intake is initially compensated by preferential expansion of subcutaneous adipose tissue (SAT), with increased adipogenesis and angiogenesis, and adipocyte hypertrophy. Subcutaneous fat acts as a “buffer” for fatty acids and triglycerides; however, this buffering capacity and the ability of SAT to expand are limited. As we demonstrated earlier, this buffering capacity depends on the proliferation potential of adipose-derived mesenchymal stem cells of SAT [29]. Once this proliferation potential is blunted, excess fat begins to accumulate in ectopic tissues and visceral depots. It is possible that individual “threshold capacities” of subcutaneous fat buffering determine the development of metabolic syndrome. For this reason, some patients develop metabolic disturbances at lower body mass and BMI values compared with those with MHO [30,31].

Obesity is commonly associated with chronic low-grade metabolic inflammation. It initially manifests in AT and gradually becomes systemic, causing insulin resistance [32]. The degree of metaflammation and the profile of pro-inflammatory mediators appear to differ between MHO and MUO [33,34,35]. MUO is characterized by higher levels of systemic inflammation compared with MHO, with significantly increased circulating pro-inflammatory factors, including cytokines and adipokines (IL-6, TNF-α, leptin, resistin, visfatin, MCP-1, PAI-1) [13,35,36,37,38,39,40], as well as greater activation of pro-inflammatory immune cells and their infiltration into adipose depots (M1 macrophages, CD8+ T cells, CD4+ Th1 cells, neutrophils, B cells) [13,35,36]. Elevated levels of CRP, leptin, and HOMA-IR observed in MUO are considered indicators of systemic metabolic inflammation [18]. The finding that individuals with MHO also exhibit signs of metaflammation, but to a lesser degree than those with MUO [35], is not uniform for all inflammatory markers (e.g., TNFα) [13]. It is important to note that there is also inconsistency between studies and that some studies found no difference in inflammatory markers between groups [41,42].

**Figure 1:**
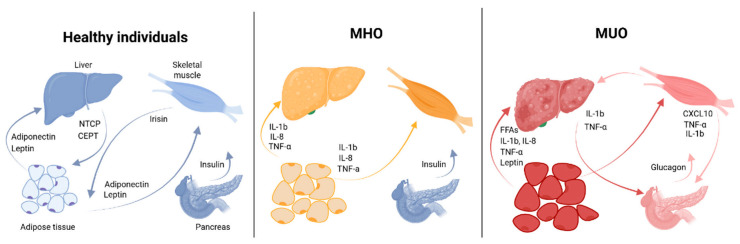
**Hypothetical scheme of metaflammation intensification that may accompany metabolic phenotype conversion**. The state of health is based upon balanced interactions between insulin-sensitive organs (AT, pancreas, liver, skeletal muscle) that regulate carbohydrate and lipid metabolism, as well as by an appropriate adaptive response to transient increases in nutrients. Key roles in the regulation of carbohydrate and lipid metabolism are played by AT hormones (leptin, adiponectin), pancreatic hormones (insulin, glucagon), myokines (irisin), and lipid metabolism mediators (cholesteryl ester transfer protein (CETP) and sodium-taurocholate cotransporting polypeptide (NTCP)). Metabolically healthy obesity (MHO) is characterized by nascent inflammation, initially triggered by AT dysfunction and the impaired regulation between insulin-sensitive organs. AT actively secretes pro-inflammatory factors (TNF-α, IL-8, IL-6, IL-1β), paving the way for systemic inflammation. Metabolically unhealthy obesity (MUO) is characterized by significant systemic inflammation, the development of systemic insulin resistance (IR), and by profound metabolic changes. An excess of TNF-α leads to impaired glucose and lipid metabolism in AT, along with hepatic inflammation, fibrosis, and IR, inducing the disturbances of glycogenolysis and gluconeogenesis. IL-6 and IL-1β contribute to the progression of metabolic-associated steatohepatitis and hepatocyte necrosis. Fatty acids, released from AT, accumulate in the liver and in muscle tissue, leading to MAFLD and myosteatosis. Ectopic fat deposition in muscle impairs normal muscle function and fatty acid oxidation, leading to mitochondrial dysfunction, oxidative stress, and inflammation, thereby reducing insulin sensitivity in skeletal muscles. Under systemic inflammation, normal pancreatic function is disrupted, with excess fat deposition and immune cell infiltration in pancreatic tissue, resulting in impaired insulin secretion by beta cells and increased glucagon secretion by alpha cells. The color gradient—blue for healthy individuals, yellow in MHO and red in MUO—reflects the intensification of metaflammation. Created in BioRender. Samsonova, M. (2026) https://BioRender.com/ll6lauu.

Although AT plays a key role in the metabolic disturbances of obesity, significant changes also occur in the liver and skeletal muscle. Visceral and ectopic (hepatic and skeletal muscle) fat distribution is a factor influencing metabolic health, possibly to a greater extent than overall fat mass [15,31]. Notably, the degree of fat accumulation in hepatocytes may even determine the obesity phenotype [43]. A number of studies report that high levels of circulating lysophosphatidylcholines (Lyso-PC), produced by the liver, can be used to distinguish between MUO and MHO, AUC = 0.77, *p* = 0.00023 [41]. There is an inverse link between Lyso-PC and CRP levels in metabolic-associated fatty liver disease (MAFLD) patients. Lyso-PC boosts regulatory T cells (Tregs), which suppress inflammation and macrophage activity, relevant for autoimmune and liver inflammation [42]. Visceral fat adipocytes can release large amounts of free fatty acids (FFAs) that reach the liver via the portal vein, leading to hepatic IR and dyslipidemia [16]. Accordingly, reduction of visceral fat volume and attenuation of metaflammation in MUO are associated with improved liver function and reduced IR [44]. Moreover, excessive hepatic fat results in MAFLD and hepatic IR, one of the key triggers in T2DM development [45]. It has been shown that individuals with MHO have a twofold increased risk of MAFLD, while those with MUO have a 3.5-fold increased risk compared with healthy non-obese individuals [46]. Skeletal muscle in MUO is also characterized by increased intramuscular fat deposition (myosteatosis), impaired fatty acid oxidation, mitochondrial dysfunction, and oxidative stress, leading to local muscle inflammation and reduced insulin sensitivity [47]. Thus, the inflammatory process in obesity involves AT, liver, and muscles, creating a vicious cycle of metabolic disturbances and promoting the MHO to MUO conversion (Figure 1).

Metabolic disturbances at MUO comprise IR with impaired carbohydrate metabolism in the form of prediabetes or T2DM, and, as a consequence, predictably higher levels of glycaemia and glycated hemoglobin when compared with patients with MHO (Table 1 and Table S1) [48,49]. In addition, patients with T2DM have a higher prevalence of concomitant dyslipidemia than those without carbohydrate metabolism disorders [50]. In T2DM, dyslipidemia is characterized by low levels of high-density lipoprotein cholesterol (HDL-C), elevated triglyceride levels, and a moderate increase in low-density lipoprotein cholesterol (LDL-C). The pathogenesis of dyslipidemia in T2DM involves increased production of very low-density lipoproteins (VLDLs) in the liver, which in turn leads to elevated triglyceride levels [51]. In association with dyslipidemia accompanying MUO, patients with T2DM develop atherosclerotic vascular disease much more frequently compared with those without T2DM. Hyperglycemia leads to increased glycation and oxidation of LDL, with their deposition in the subendothelial space; oxidative stress; increased formation of advanced glycation end products (AGEs) with activation of the intracellular NF-kB signaling pathway; reduced nitric oxide (NO) production; and increased angiotensin II levels, as well as recruitment of monocytes/macrophages into the subendothelial space and their release of pro-inflammatory cytokines. This results in endothelial dysfunction and inflammation and enhanced formation of atherosclerotic plaques [52]. Some clinical, metabolic and AT differences between the MHO and MUO are summarized in Table 1 and Table S1.

## 3. The Role of Low-Grade Inflammation in the Development of Obesity

Although the association between obesity and inflammation was established back in the 1990s by Gökhan S. Hotamisligil et al. [32], the exact mechanisms of their mutual influence and subsequent aggravation are still an issue. Obesity-associated metaflammation is chronic, does not resolve within normal timeframes, and causes constant recruitment of immune cells to AT [63], involving metabolic tissues into the pathological process and leading to their dysfunction [64].

Based on the observations and clinical data mentioned above, it can be noted that individuals with MHO exhibit moderate systemic and AT inflammation (ATI), which allows them to maintain metabolic health. In contrast, inflammation in MUO reaches a higher intensity, resulting in the development of a range of metabolic disorders. In this regard, several authors have put forward the hypothesis that MHO represents an intermediate phenotype between metabolically healthy non-obese individuals and MUO (Figure 1) [3,4,15,35]. Notably, AT dysfunction is one of the key elements in the development of systemic chronic inflammation and metabolic disturbances. Normally, AT serves as an energy reservoir in the form of triglycerides and functions as an endocrine organ, releasing hormones and bioactive substances (adipokines) that regulate numerous physiological processes related to energy balance, glucose metabolism, and immunity [65]. Cytokines produced by adipocytes and immune cells in AT may exhibit both pro-inflammatory and anti-inflammatory properties, thereby increasing or reducing the level of IR.

The initial stage of AT metaflammation remains an issue; however, it is believed that adipocyte hypertrophy and hypoxia in VAT, LPS, the lipopeptides derived from bacteria, and endogenous lipids contribute to the increased expression of proteins like TLRs, NF-kB, and HIF1α that are associated with inflammation [66]. Interestingly, the AT volume increases with age primarily because of adipocyte hypertrophy rather than hyperplasia [67]. It is precisely hypertrophied adipocytes that are more prone to activating endoplasmic reticulum (ER) and mitochondrial stress responses, promoting the initiation of a chronic pro-inflammatory state in AT [68]. ATI is accompanied by adipocyte apoptosis, the release of chemotactic mediators, and leukocyte inflammatory infiltration of the fat. Profound changes occur in the composition of resident and migrating immune cells.

In healthy AT, anti-inflammatory immune cells predominate, including M2 macrophages, invariant NKT (iNKT) cells, group 2 innate lymphoid cells (ILC2s), eosinophils, regulatory T cells, and type 2 helper T lymphocytes. In individuals without excess weight and metabolic syndrome, adipocytes predominantly secrete anti-inflammatory cytokines such as adiponectin, transforming growth factor beta (TGF-β), IL-10, IL-4, IL-5, IL-13, neuregulin 4, IL-1 receptor antagonist (IL-1Ra), and apelin. Thereby they maintain the homeostasis of healthy AT and prevent the accumulation and activation of pro-inflammatory immune cells [36,69,70]. In response to altered metabolism and AT stress, the levels of FFAs, palmitic acid, and ceramides increase, along with neutrophil recruitment to AT [71]. Persistently maintained local inflammation leads not only to the accumulation of immune cells but also to the shift in their profile: pro-inflammatory cells begin to predominate—the proportion of mast cells, B cells, NK cells, type 1 helper T lymphocytes, cytotoxic CD8+ T cells, ILC1, and macrophages polarizing towards the M1 phenotype in the presence of NK cells increases. An increase in the level of circulating gut antigens can also contribute to the initiation and development of ATI. In individuals with obesity, and especially with MUO, AT predominantly secretes pro-inflammatory cytokines (Table 2) together with obesogenic adipokines, including leptin, visfatin, chemerin, vaspin, resistin, angiotensin II, and plasminogen activator inhibitor-1 [72]. Conversely, levels of anti-inflammatory IL-4, IL-5, IL-10, IL-13, adiponectin, and omentin are reduced in MUO [15,65].

Abundant pro-inflammatory mediators and the increasing number of migrating pro-inflammatory immune cells within AT contribute to its further dysfunction and damage. This leads to the emergence of additional so-called second-order pro-inflammatory molecules, most of which are associated with damage-associated molecular patterns (DAMPs) and are frequently observed in MUO—such as succinate [98], N-formyl peptides [99], AGEs, cholesterol crystals, islet amyloid peptide [100], and reactive oxygen species (ROS). Monocytes/macrophages, key mediators of inflammation, are capable of recognizing more than 1000 DAMP patterns [101]. Patients with MUO exhibit more pronounced infiltration of AT by macrophages and other immune cells, as well as a higher density of crown-like structures [102]—apoptotic adipocytes surrounded by macrophages, which serve as markers of ATI and dysfunction [103]. Prolonged stimulation by obesogenic antigens leads to the appearance of memory cells in inflamed tissues and alters their epigenetic profile. Senescent cells, particularly among T cells, emerge, producing a distinctive pro-inflammatory profile, known as the senescence-associated secretory phenotype (SASP). This includes IL-6, IL-1β, MCP-1, PAI-1, IL-8, insulin-like growth factor binding protein 3 (IGFBP3), high-mobility group protein B1 (HMGB1), and ROS. Several of these mediators are also associated with MUO—HMGB1 [104], IGFBP3 [105], and PAI-1 [82]. Subsequent AT dysfunction, including destabilization of angiogenesis, remodeling impairment and adipogenesis attenuation, ultimately lead to both local and systemic IR [67,106].

Pro-inflammatory adipokines modulate IR of adipocytes either directly, by affecting the insulin signaling pathway, or indirectly, through stimulation of other inflammatory pathways. The phosphorylation of serine residues in the insulin receptor substrate (IRS) molecule by various adipokines, either directly or via inflammatory pathways including the c-Jun N-terminal kinase (JNK) pathway and the NF-κB pathway, impairs signal transduction from the insulin receptor, leading to local IR [65].

Thereafter, dysfunctional AT plays a primary and central role in the initiation and progression of subsequent systemic inflammation. Pro-inflammatory mediators released by AT into the circulation enhance hematopoiesis (both lymphopoiesis and myelopoiesis), attracting increasing numbers of various leukocyte types into the bloodstream and into adipose and other tissues, thereby promoting a more widespread inflammation [107]. Patients with MHO exhibit an intermediate phenotype, characterized by a balance between pro- and anti-inflammatory mediators [108]. Metaflammation accompanying obesity begins to spread beyond AT, developing in other organs (liver, muscles, pancreas) involving not only recruited and activated immune cells but also myokines (in muscles) and hepatokines (in the liver). Visceral fat releases a large amount of pro-inflammatory cytokines and FFAs in MUO. The release of FFAs into the bloodstream causes hepatocyte injury and stimulates the synthesis of pro-inflammatory cytokines. IL-17 promotes cytokine production by adipocytes and macrophages, contributing to the development of MAFLD, including steatohepatitis, impaired liver function, and hepatocyte death [109]. As in AT, macrophages accumulating in the liver in obesity are characterized by an M1 phenotype. Excessive triglyceride accumulation and hepatic inflammation are accompanied by increasing hepatic IR [110]. Recent studies also demonstrate the presence of obesity-related inflammation in the central nervous system and gastrointestinal tract [111,112]. It is important to note that prolonged exposure to pro-inflammatory stimulation underlying obesity and metaflammation induces epigenetic changes and functional exhaustion of cells. This functional exhaustion exacerbates the severity of inflammation in all metabolically active tissues and leads to dysfunction of both mature and progenitor cells [113].

**Figure 2:**
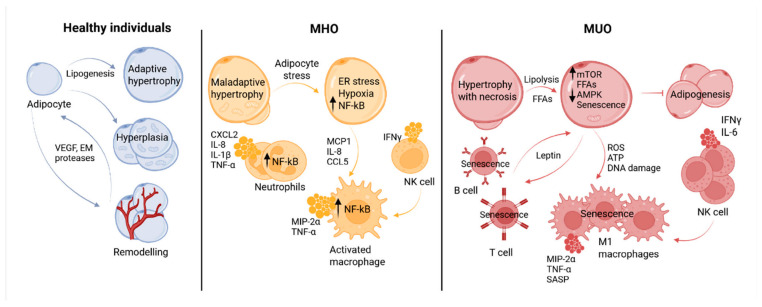
**The plausible extrapolation model of inflammation progression in AT that may accompany the conversion from MHO to MUO in a worse-case scenario.** Normally, short-term nutrient excess elicits a physiologically adaptive response, aimed at removing lipid overload and excessive glucose from the bloodstream to maintain metabolic homeostasis. This involves moderate adipocyte hypertrophy, preadipocyte differentiation, and AT remodeling. The **MHO** stage is characterized by the activation of the NF-κB pathway in hypertrophic adipocytes due to hypoxia and endoplasmic reticulum stress, leading to the secretion of pro-inflammatory mediators (IL-6, IL-8, CCL5, PAI-1), which attract neutrophils and other blood leukocytes to AT. The immune cell profile shifts from anti-inflammatory to pro-inflammatory Th1/M1 type. Leptin, which increases with the progression of obesity, stimulates lipolysis and activates its own receptors on immune cells, promoting their activation and metabolic reprogramming. Expression of MHCII and the production of co-stimulatory molecules in adipocytes further activate T cells. The increased concentration of FFAs due to lipolysis, in turn, reactivates macrophages, neutrophils, and adipocytes. The **MUO** stage is typified by an exacerbation of inflammation, as well as functional and structural changes in tissues. In response to DNA damage, mitochondrial dysfunction and ROS generation, AT secretes additional immunostimulatory mediators associated with further tissue damage (multiple DAMPs, stress proteins, HMGB1, DNA, mitochondrial components). Cells with a senescent phenotype (macrophages, endothelial cells, preadipocytes, and mature adipocytes, T cells) increasingly accumulate in AT. The color gradient—blue for healthy individuals, yellow in MHO, and red in MUO—reflects the intensification of metaflammation. Abbreviations: VEGF—vascular endothelial growth factor; ECM—extracellular matrix; FFAs—free fatty acids; ER—endoplasmic reticulum; NF-κB—nuclear factor kappa-light-chain-enhancer of activated B cells; MCP-1—monocyte chemoattractant protein 1; CCL5—chemokine (CC motif) ligand 5; PAI-1—plasminogen activator inhibitor-1; HMGB1—high-mobility group protein B1; ROS—reactive oxygen species; MHCII—major histocompatibility complex class II; mTOR—mechanistic target of rapamycin; AMPK—AMP-activated protein kinase; ATP—adenosine triphosphate; IL—interleukin; DAMPs—danger-associated molecular patterns. Created in BioRender. Samsonova, M. (2026) https://BioRender.com/ek230nz.

A possible mechanism underlying the intensification of inflammation and AT dysfunction during obesity progression is illustrated in Figure 2. Though the conversion of MHO into MUO may proceed through escalation of local and systemic inflammation, the exact mechanism and precise biomarkers of the MHO progression remain unclear and vary between studies. It is well recognized that MHO is not a uniform condition, it differs in severity. Such factors as higher insulin sensitivity, functional SAT, lower accumulation of visceral and liver fat, younger age, high cardiorespiratory fitness and physical activity contribute not only to MHO stability, but also to its heterogeneity [7]. Not every individual transforms his MHO into MUO [114]. This adds complexity to the identification of the precise risk factors of MHO transition into morbid MUO.

## 4. Differences in the Levels of Circulating Pro-Inflammatory Factors Between Metabolically Healthy Obesity (MHO) and Metabolically Unhealthy Obesity (MUO)

Literature analysis reveals that progression from MHO to MUO can be accompanied by increasing level of circulating pro-inflammatory factors (IL-6, IL-8, IL-17, IL-21, IFN-γ, TNF-α, MCP-1, CRP, FFAs, advanced glycation end-products (AGEs), cell adhesion molecules, leptin, chemerin, resistin, etc.) together with a reduction in the level of anti-inflammatory cytokines (IL-4, IL-5, IL-10, IL-13) and adipokines (adiponectin, omentin) [115]. Representative data on the level of circulating mediators and their changes in the conditions of MHO and MUO are illustrated in Table 2. Most studies were cross-sectional in design, whilst longitudinal investigations spanning 10–30 years within a single MHO cohort in order to validate biomarkers of the transition to MUO are exceptionally difficult to conduct. Consequently, such studies are exceedingly rare [75,77,85]. On the other hand, the lack of standardization in the definition of MHO poses challenges for performing meta-analyses, although meta-analyses are nevertheless encountered for certain aspects, such as CRP, IL-6, TNF-α [13]. According to meta-analysis, CRP and IL-6 levels significantly differ between groups and can distinguish conditions of MHO and MUO. Notably, meta-analysis revealed an increase in TNF-α (which may be secreted by stressed adipocytes and macrophages) levels in MHO compared with healthy lean group, while detecting no significant differences between MHO and MUO [13].

Stressed cells of AT in obesity may secrete different profiles of mediators in MHO and MUO. MHO children display more favourable adipokine profiles than MUO children, with lower leptin, higher adiponectin [97]. RBP-4 and leptin/adiponectin ratio (OR: 0.58; 95% CI) was reported as an independent predictor of MHO in children [96].

In MUO, insulin-resistant AT is characterized by a predominance of basal and catecholamine-induced lipolysis, leading to elevated FFA plasma levels, which are associated with IR and beta-cell dysfunction [116,117]. Consequently, T2DM is often accompanied by increased plasma FFAs [118,119]. Lyso-PC might be a precise biomarker of MUO to separate metabolically benign from malignant NAFL [41], while the other paper showed a reduction of plasma Lyso-PC in obese, with no differences between MHO without T2DM and MUO with T2DM [42].

In recent years, AGEs and their role in the pathogenesis of chronic inflammation and metabolic complications in obesity have been actively studied. Advanced glycation end-products constitute a non-homogenous, chemically diverse group of compounds formed either endogenously, especially in the context of pre-existing metabolic disorders, or exogenously, particularly with sugar-sweetened beverages/foods, through smoking, etc. Metabolic syndrome, including obesity, hyperlipidemia, and hyperglycemia, can be accompanied by enhanced AGE production via the non-enzymatic Maillard reaction between reducing sugars (glucose, fructose, galactose, ribose, or deoxyribose) and various molecules (proteins, lipoproteins, lipids, or DNA). Upon binding to their receptors (RAGE), various AGE products can induce inflammation, IR, T2DM, and diabetic complications [120].

Cell adhesion molecules are upregulated under conditions of chronic hyperglycemia and oxidative stress and facilitate the transmigration of leukocytes into tissues, promoting chronic inflammation, endothelial dysfunction, and micro- and macrovascular complications in T2DM. The main cell adhesion molecules involved in the development of microvascular complications are vascular cell adhesion molecule-1 (VCAM-1), intercellular adhesion molecule-1 (ICAM-1), and selectins (E-selectin, L-selectin, and P-selectin). Follow-up (2 years study) indicate that levels of cell adhesion molecules are elevated in patients with T2DM and their growth is associated with complications: P-selectin (*p* = 0.05) and L-selectin (*p* = 0.008) with nephropathy, while retinopathy is associated with L-selectin (*p* = 0.005) only [121].

Thus, the changes observed in MUO are characterized by chronic hyperglycemia, pronounced dyslipidemia, proatherogenic status, endothelial dysfunction, enhanced formation of AGEs and elevation of lipolytic activity accompanied by the release of FFAs. At the same time, such factors as variations in definition of MHO, many various complications characterized for MUO, and rare data of meta-analysis make it difficult to clarify the universal and exact biomarkers of MHO/MUO transition. Although numerous parameters listed in Table 2 exhibit significant differences in certain studies, only CRP and IL-6 fulfil the criteria for a validated biomarker.

## 5. Differences in the Number and Activity of Circulating Leukocytes and Their Subpopulations

Circulating leukocytes represent a heterogeneous group of cells that participate not only in the defense against foreign agents but also in the elimination of aberrantly functioning cells (due to stress or tumor transformation) and their degradation products. As a component of the immune system and the primary source of immune cells infiltrating AT during obesity, blood leukocytes and their subpopulations could potentially serve as biomarkers of subclinical inflammation and predictors of morbid obesity [57,122]. Indeed, there are studies that report a trend towards an increase in the total leukocyte counts in obesity and metabolic disorders [123,124,125]. However, the absolute count of all leukocytes or their major subsets (neutrophils, eosinophils, basophils, monocytes, lymphocytes, and their main subtypes—T, B, and NK lymphocytes) lacks clinical significance to reveal patients at risk of converting from MHO to MUO [122]. The range of leukocyte counts, both total and for their individual subpopulations, often falls within normal reference intervals, and is characterized by high variability with overlapping values between comparison groups. This complicates the determination of clinically meaningful cut-off values (Table 3). The clinical utility of circulating leukocytes counts (and their major subtypes) is limited, likely because most studies are cross-sectional and do not assess this parameter dynamically. Notably, longitudinal studies indicate that a baseline leukocyte count of 6.5–6.9 × 10^9^ cells/L may serve as an independent predictor of diabetes development, even in non-obese patients [57,123,126].

In a large cohort (24,897 individuals from the Metabolic, Lifestyle and Nutrition Assessment in Young Adults (MELANY) cohort), divided into five groups by white blood cell count and followed for an average of 7.5 years, a direct correlation was found between new cases of T2DM and the baseline leukocyte count, independent of other factors (age, heredity, physical activity, and even obesity (BMI). Adjusting for obesity significantly reduced the predictive power of the total leukocyte count in the model but still retained it as a significant factor [126]. Patients with obesity and elevated white blood cell count (>6.5 × 10^9^ cells/L) are at higher risk of developing T2DM [123]. Another study also found that an elevated baseline leukocyte count correlated with the development of T2DM and insulin action worsening [14].

Monocytes play an important role in the pathogenesis of obesity, which is often associated with an increase in their number in the circulation [122,137,138]. Moreover, after the discovery of monocyte and macrophage infiltration into the AT of mice and humans, circulating monocytes have attracted considerable attention [139,140]. Monocytes are known to play a key role in the pathogenesis of atherosclerosis, a complication of obesity [141,142]. Activation of myelopoiesis, and monopoiesis in particular, has been reported in animals and humans on a high-calorie diet [143,144]. However, data on changes in the number of circulating monocytes in different obesity phenotypes are conflicting. Higher blood monocyte levels (on average 13–18% higher) have been recorded in overweight and obese individuals compared with those without excess weight, and are correlated with higher BMI values and metabolic syndrome [122,137,138,145]. Nevertheless, other studies have not found such a correlation [146,147]. No consistent patterns of change in the number of classical CD14+CD16- monocytes, which constitute the majority of monocytes and reflect changes in their total population, were found either [122,146]. On the other hand, a higher frequency of circulating CD16+ monocytes in obesity and their correlation with BMI and waist circumference have been reported—individuals with obesity had almost a doubling in CD16+ monocytes compared with 4.1 ± 1.7% in lean individuals [137,145,148]. Notably, correlations have been observed between systemic inflammation (the number of CD16+ monocytes) and inflammation in SAT (the number of CD68+ macrophages) [148], as well as between an increase in circulating non-classical monocytes (CD14+CD16++) and lipid accumulation in VAT macrophages [149] in obese patients compared with healthy controls. However, it is unlikely that specific circulating monocyte subpopulations could be direct precursors to specific macrophage subpopulations in inflamed AT [122,149]. It is possible that the activation status of monocytes more accurately captures the alterations associated with metabolic dysregulation in obesity. Indeed, increased expression of the integrin molecule CD11b is observed in obesity, and correlates with IR, per Table 3 [56].

Neutrophils are the most abundant group of blood leukocytes. They play a crucial role in the early stages of inflammatory responses and represent the primary line of defense during inflammation. As cells with cytotoxic functions, they are the first to migrate to the site of inflammation, preceding the arrival of monocytes/macrophages, dendritic cells, and various types of lymphocytes [71]. Neutrophils exert their effector functions through (i) the oxidative burst and release of lytic enzymes (myeloperoxidase and elastase); (ii) phagocytosis and the production of pro-inflammatory cytokines; and (iii) the formation of neutrophil extracellular traps (NETs), thereby eliminating the source of inflammation and subsequently undergoing apoptosis. Notably, obesity-associated inflammation is also accompanied by an increase in circulating neutrophils [122,150]. A correlation is reported between neutrophil levels and waist circumference as well as BMI. Overweight patients with neutrophilia demonstrated elevated CRP levels [124]. In the large PREDIMED study, higher baseline neutrophil levels or an increase in neutrophil count over more than 3.5 years of follow-up were independently associated with an approximately 30% increased risk of developing MUO among individuals aged 55 years and older without cardiovascular disease [138]. In addition, neutrophils in MUO are characterized by an activated phenotype, with elevated levels of myeloperoxidase and calprotectin and increased expression of CD66b, a marker of neutrophil degranulation [94].

The neutrophil-to-lymphocyte ratio (NLR), a well-known marker of systemic inflammation, may serve as an early indicator and predictor of clinical complications in T2DM, such as nephropathy, retinopathy and macular oedema, ketoacidosis, and diabetic foot syndrome [151]. Moreover, an elevated NLR can reveal ongoing subclinical inflammation in MHO [128,152], while a high NLR is a statistically robust and independent predictor of T2DM in MUO, per Table 3 [127].

Eosinophils, known for their role in regulating allergic reactions and responses to parasitic infections, can infiltrate specific tissues, where they play an additional role in regulating local immunity and/or tissue remodeling and repair [153]. The protective role attributed to eosinophils in obesity appears to be played by AT eosinophils rather than circulating eosinophils [154]. Data on changes and the function of circulating eosinophils are scarce and contradictory. Some studies have reported increased absolute and relative eosinophil counts, an association between obesity and eosinophilia, and a positive correlation between eosinophil count, BMI, and metabolic syndrome [155]. Other studies have not found changes in eosinophil levels [156]. One study notes an overall positive correlation between eosinophil count and BMI, but after eosinophils exceed 200 cells/μL, this correlation becomes negative [157].

Although T lymphocytes are not critical drivers of metaflammation, they can significantly modulate it [158]. Various T cell subpopulations release cytokines, sustaining inflammation in both MHO and MUO. Moreover, T cells, both in the periphery and locally in AT, may indicate changes in metabolic status from prediabetes to T2DM [159]. Some studies have found that the total number of T cells increased by 15–50% in individuals with obesity compared with those without excess weight [160], and this increase was associated with specific components of metabolic syndrome (higher triglyceride levels, lower HDL cholesterol) [129].

Analysis of lymphocyte subpopulations is sometimes contradictory. Regarding CD4+ T cells, most researchers report elevated levels in both MUO [160] and MHO [161] compared with non-obese individuals. CD4+ T cells are coordinators of the immune response. Depending on the antigen, dose and duration of stimulation, on the co-stimulatory chemokines/cytokines in the microenvironment, and on interactions with other cells, CD4+ T lymphocytes differentiate into several main subtypes (Th1, Th2, Th9, Th17, Th22, follicular T cells, and various subsets of FoxP3+ regulatory T (Treg) lymphocytes), directing the immune response according to distinct cytokine secretion profiles [162]. Th1 lymphocytes produce IFN-γ and IL-2, with their differentiation driven by IL-12 [163]. Th2 lymphocytes are associated with the humoral immune response and secrete anti-inflammatory cytokines IL-4, IL-5, IL-9, and IL-13, stimulating the activation of eosinophils and M2 macrophages [164]. Th17 cells are characterized by secretion of IL-17, IL-21, IL-22, and granulocyte-macrophage colony-stimulating factor (GM-CSF), whereas regulatory T cells produce IL-10 and TGF-β [165].

Several studies have demonstrated that the number of Th1 cells in peripheral blood does not differ between individuals with obesity and those without [160]. However, other authors report that the percentage of Th1 cells does increase in patients with obesity and correlates with the degree of IR [56]. Interestingly, the number of circulating Th2 cells, which have an anti-inflammatory profile, also increases in individuals with pathological obesity and correlates with BMI [160]. Nevertheless, the Th1/Th2 ratio remains higher in MUO compared with MHO and non-obese individuals, per Table 3 [56]. In individuals with obesity and T2DM, elevated levels of circulating Th1 and Th17 lymphocytes positively correlate with IR, leptin and insulin levels, and negatively with HDL cholesterol [166]. In addition to changes in the number of Th subtypes and their ratios, Th cell functional activity is enhanced. This is manifested by increased cytokine production and a shift towards a pro-inflammatory profile, even in MHO [13,133,158]. Increased Th1 and Th2 cytokines in obesity are often a marker of prediabetes [159]. The production of pro-inflammatory cytokines by CD4+ T cells is higher in MUO compared with MHO [80]. MHO is characterized by the presence of Th2 cytokines IL-10, IL-13, and IL-4 [167]. In contrast, MUO with T2DM is characterized both by Th1 cytokines IL-2 and IL-12p70, and Th17 cytokines IL-17A, IL-17F, IL-21, IL-22, MIP3α/CCL20, and GM-CSF, the majority of which are produced by Th17 cells [80]. In obesity, Th17 cells make a particular contribution to inflammation and hyperglycemia. IL-17 may play an important role in recruiting neutrophils and macrophages.

Obesity, as a chronic inflammatory disease, is closely linked to immunosenescence [168]. In the study by Sbierski-Kind et al., MHO with an insulin-sensitive phenotype was found to be associated with a higher proportion of naïve CD4+ and CD8+ T cells, while MUO with an insulin-resistant phenotype positively correlated with the frequency of CD45RA-CD27-CD28- effector and CD57+ senescent CD8+ memory T cells [169]. Increased levels of senescent CD4+ and CD8+ T cells with the CD45RA+CCR7- and CD45RA+CD28-CD27-CCR7-KLRG1high phenotype have also been observed in T2DM [170,171].

Regulatory FoxP3+ T cells (Tregs) are a subset of T helper cells whose unique role is to maintain immune tolerance and suppress the activation, proliferation, and effector responses of innate and adaptive immune cells. In contrast to Th1, Th17, Th2, and Th9 (effector) cells, which promote systemic and local inflammation in AT during obesity, Tregs counteract the development of inflammation [159]. Patients with obesity show an inverse correlation between BMI and the proportion of circulating Tregs [172]. It has been reported that the percentage of circulating Treg cells among total CD4+ T cells is lower in individuals with obesity compared with those without excess weight [58], being lower in MUO than in MHO. The percentage of Tregs may be an indicator of metabolic health: a lower number of Treg cells is associated with higher leptin and HbA1c levels, i.e., with MUO [58].

FoxP3+ Tregs represent a highly heterogeneous group of cells. Primarily, they are categorized into two main groups based on their origin: natural Tregs (nTregs) that develop in the thymus, and induced Tregs (iTregs), which arise in the periphery under the special microenvironment [173]. Naïve CD4+ T cells can differentiate into FoxP3+ iTregs, particularly in the presence of TGF-β and low-dose antigenic stimulation. Interestingly, under certain conditions, many differentiated Th cells demonstrate a high degree of plasticity, changing their phenotype and function. The conversion of Tregs into Th1-like cells has been described; these cells co-express the transcription factors FoxP3 and Tbet and secrete IFNγ. The emergence of such Th1-like Tregs has been observed in some autoimmune diseases, including type 1 diabetes [174]. Furthermore, the plasticity between Tregs and Th17 cells is also well recognized and characterized by reciprocal relationships. Under certain conditions, in the presence of IL-1β or under the influence of microbial stimulation of TLR2 or epigenetic modifications, FoxP3+ Treg cells can differentiate into Th17-like Tregs expressing IL-17 [175]. The balance between pro-inflammatory Th17 and anti-inflammatory Tregs is critical for maintaining immune homeostasis, as it determines whether the immune response develops towards immunosuppression or inflammation. Numerous endogenous microenvironmental factors, including cytokines and metabolic factors, regulate the differentiation of Th17 and Tregs arising from naïve CD4+ T cells [176]. Thus, TGF-β, which is important for the differentiation of both Th17 and Tregs, in the presence of pro-inflammatory cytokines (IL-6, IL-1β), directs the differentiation of iTregs towards Th17 lineage. Cell activation accompanied by metabolic reprogramming can also influence differentiation. Chronic excess of glucose and lipids directly impacts the activation of Th17 and Tregs, modulating the activity of energy sensors, influencing cellular metabolism [176]. Specifically, Th17 cell function depends on glycolysis, fatty acid synthesis (FAS), and glutaminolysis, whereas Tregs rely on oxidative phosphorylation (OXPHOS) and fatty acid oxidation (FAO) [176]. Activation of the mammalian target of rapamycin (mTOR), a key regulator of cellular metabolism, is necessary for the differentiation of effector T cells, particularly Th17. mTOR is activated in response to PI3K/Akt pathway activation following the activation of naïve T cells after TCR stimulation and the receipt of a co-stimulatory signal. mTOR stimulates hypoxia inducible factor 1α (HIF1α), which regulates the transcription of many glycolytic enzymes. HIF1α is required for Th17 T cell differentiation and inhibits Tregs [177]. Inhibition of mTOR by rapamycin shifts Th cell differentiation towards Tregs. A high-calorie diet promotes differentiation of the Th17 phenotype via activation of FAS de novo, mediated by acetyl-CoA carboxylase 1 (ACC1). This leads to increased expression of IL-17A, IL-23R, leukotriene B4 receptor (LTB4R), and CCR6. AMPK, an energy deficiency sensor activated by an increased AMP/ATP ratio, inhibits mTOR activity and promotes FoxP3-induced OXPHOS and FAO, enabling cells to produce ATP and generate energy. AMPK is particularly active in Tregs, and its activation leads to the differentiation of naïve T cells into Tregs rather than Th17, enhancing FAO. Notably, metformin, an indirect activator of AMPK, suppresses T cell proliferation and inhibits Th17 differentiation, thereby promoting Tregs [178]. Thus, the balance between pro-inflammatory Th17 and anti-inflammatory Tregs may play a critical role in the development of obesity and the transition from MHO to MUO.

The data on cytotoxic CD8+ T cells are contradictory, but most studies report a decrease in the absolute and/or relative content of CD8+ T cells in obesity, especially in MUO [20,122]. However, among CD8+ T cells, an increase in the number of effector memory IL-7Rα^low^CX3CR1^+^ CD8+ T cells was observed in children with MUO reaching, 53.8 ± 20.1% vs. 41.5 ±11.9%, *p* = 0.036 [179].

B cells also contribute to the development of metabolic dysfunction. Through their ability to (1) produce antibodies, (2) present antigens and regulate T cell responses, and (3) secrete cytokines, B cells can control obesity-associated inflammation by modulating cytokine production by T cells [180]. Improvement of metabolic disease following B cell depletion in animal models suggests that B cells may play a pathogenic role [181]. According to research data, the number of B lymphocytes in the blood is increased in individuals with obesity compared with those with normal weight [122]. Elevated circulating B lymphocyte levels (>14.95%) were independently associated with increased risk of developing T2DM, suggesting a potential link between MHO and B cell-mediated inflammation [182].

Obesity is also accompanied by changes in the B cell subpopulations. Normally, B cells consist of naïve IgD+CD27- B cells, memory B cells with switched (IgD-CD27+) and unswitched (IgD+CD27+) antibody classes, and a small proportion of double-negative IgD-CD27- (DN) B cells. In obesity, the proportion of switched IgD-CD27+ memory B cells decreases, while the frequency of DN B cells increases. These DN B cells, also known as late memory B cells, are rendered as an inflammatory B cell subpopulation [183]. Notably, circulating B cells isolated from individuals with obesity secrete elevated (three-times higher) levels of IgG reactive to self-protein lysates from SAT [184]. These B cells, which secrete antibodies against self-antigens, are characterized by CD95+CD21-CD11c+ phenotype and expression of the transcription factor T-bet [185]. Furthermore, the balance of pro- and anti-inflammatory B cell subpopulations in individuals with obesity is shifted towards a reduction in anti-inflammatory regulatory CD19+CD24highCD38high B cells (Bregs), which secrete IL-10 (in both MUO and MHO) [186,187]. Obesity is also associated with a decrease in the peripheral transitional CD19+CD27+CD38high B cells [130], among which IL-10-secreting and CD1d-expressing Bregs may constitute a significant fraction. B cells isolated from the blood of individuals with obesity secrete IL-6 and TNF-α more actively, and IL-10 less actively, after in vitro stimulation [185].

The pro- and anti-inflammatory subpopulation balance of B cells may be regulated by an individual’s metabolic status—in particular, through interaction with leptin. Leptin, highly elevated in obesity [88], interacts with Ob-Rb receptors on B cells and stimulates protein synthesis and ribosome biogenesis through mTORC1 activation, while inhibiting autophagy. Activation of this pathway in immune cells is generally associated with pro-inflammatory function [130].

Chronic obesity-associated inflammation leads to an imbalance in the structure of all lymphocytes, including B lymphocytes, and results in a weaker immune response to infections, vaccination, or tumors. Normally, B cell response develops through early extrafollicular activity, subsequent induction of a response in germinal centers (GC), and culminates in final production of high-affinity and long-lived antibodies and the formation of memory B cells. In obesity, B cell activation occurs mainly extrafollicularly, reducing the availability of precursors for GC induction. This enhances the production of autoantibodies by IgD-CD27- DN B cells, and leads to a reduction in GC-derived plasma cells and memory cells capable of producing high-affinity neutralizing antibodies [186]. As a consequence, this provokes a severe course of infectious disease in MUO due to the uncontrolled extrafollicular activity and formation of low-affinity antibodies with reduced neutralizing activity and a narrow range of epitopes against foreign antigens. Additionally, it is exacerbated by a reduced proportion of Bregs and IL-10-secreting transitional B cells, concomitant with the expansion of IL-6- and TNF-α-producing B cells.

NK cells attract particular attention in obesity studies due to their involvement in the innate immune response to infections and malignancies, the incidence of which increases with the severity of obesity and its morbid phenotype [188]. In addition, NK cells are an important link between obesity-associated metabolic stress and inflammation in VAT [189]. The previous studies demonstrate a complex and contradictory picture regarding changes in circulating NK cells and their functional activity in obesity, with the prevailing concept of a reduction in NK cell count in MUO and no changes in MHO [20,122,131]. Some studies report that patients with obesity generally exhibit reduced circulating NK cells compared with healthy, non-overweight individuals, with MUO patients having nearly 50% fewer CD56+ NK cells than MHO patients [20]. However, in children with obesity and hepatic steatosis, the absolute NK cell count was 66% higher than in obese children without steatosis [122]. A number of studies report no differences between healthy and overweight individuals [188,190,191]. Among the general NK cell population, some researchers demonstrate a decrease in CD56^dim^CD16^bright^ and a three-fold increase in CD56^bright^CD16^dim/neg^ NK cell subpopulations in obesity [133]. This could be caused by the activation of NK cells by obesity-associated metabolic products and conversion of the CD56^dim^CD16^bright^ subpopulation to the CD56^bright^CD16^dim/neg^ phenotype [188]. Metabolic changes in obesity also affect the functional properties of NK cells, but the data are conflicting. Both increased activation (increased expression of CD69, NKp46 [192]) and decreased activation of NK cells (reduced proportion of CD69, NKp46, and NKG2D-positive cells [133,192]) have been reported in obesity. There are frequent reports on the reduced secretion of granzyme B, perforin and macrophage inflammatory protein (MIP)-1β by NK cells from obese people during interaction with tumor cell lines in vitro [192]. This could be one of the reasons for the increased risk of tumor formation in obesity. Interestingly, a 3-fold increase in the specific population of NK cells expressing IL-6 receptors (IL-6Ra) and Csf1r (colony stimulating factor 1 receptor) has been described in humans and mice. This subset accompanies metaflammation and IR. IL-6/STAT3-dependent formation of this NK cell subset correlates with MUO [190].

Based on the data presented above, stratification of patients with different obesity phenotypes according to altered profiles of circulating leukocytes is not yet standardized in clinical practice. Threshold values for various leukocyte subpopulations to predict MUO and T2DM have not been fully established. At the same time, changes in some parameters are consistently observed in several studies and correlate with MUO development. There is a general trend towards increased total numbers of leukocytes, lymphocytes, neutrophils, monocytes, B lymphocytes (particularly IgD-CD27- DN B cells, or late memory B cells), CD4+ T lymphocytes, as well as Th1 and Th17 lymphocytes. The ratio of pro- to anti-inflammatory cells may play a significant role—MUO is often accompanied by an increased neutrophil/lymphocyte ratio (NLR), as well as Th1/Th2 and Th17/Treg ratio. In addition, MUO positively correlates with the frequency of senescent CD4+ and CD8+ T cells with the CD45RA+CCR7- and CD45RA+CD28-CD27-CCR7-KLRG1^high^ phenotypes, and with CD45RA-CD27-CD28- effector and CD57+ senescent CD8+ memory T cells, which secrete pro-inflammatory cytokines. MUO is also associated with the emergence of a specific population of IL-6+/STAT3+ NK cells and an increase in CD56^bright^CD16^dim/neg^ NK cells. At the same time, the transition from MHO to MUO is marked by a decrease in the number of Tc-cytotoxic, Treg-regulatory, regulatory CD19+CD24^high^CD38^high^ B cells (Bregs), CD19+CD27+CD38^high^ B cells, switched IgD-CD27+ memory B cells, and CD56^dim^CD16^bright^ NK cells. In summary, the absolute numbers of leukocytes and neutrophils are the most statistically significant and validated parameters correlating with MUO in longitudinal studies, with an AUC of no more than 0.6.

## 6. Cellular Differences in the Adipose Tissue Associated with Metabolically Healthy Obesity (MHO) and Metabolically Unhealthy Obesity (MUO)

Numerous studies have demonstrated that obesity-associated changes in immune cell balance occur in AT. It is well established that obesity triggers a more complex and intense inflammatory response in VAT than in SAT, and it is precisely the cellular changes in visceral fat that are more strongly associated with MUO [193]. Notably, it has been reported that insulin sensitive obesity, as observed in MHO, has significantly lower ATI [90,194].

Neutrophils. As the first cells to infiltrate AT, neutrophils play a key role in the early stages of obesity and its associated inflammation [71]. Normally, when migrating into healthy AT in response to an inflammatory stimulus, neutrophils do not remain there for long. Macrophages that arrive in their place, and which polarize towards the anti-inflammatory M2 phenotype, clear away apoptotic neutrophils and damaged tissue via the process of repair and tissue remodeling. Any disruption of this tightly coordinated response and failure to resolve inflammation within a short time window leads to chronic inflammation, contributing not only to secondary tissue damage but also to immune exhaustion and aging, as observed in metabolic disorders [195]. However, animal models have shown that, in diet-induced obesity, neutrophils persist in AT for extended periods (over 90 days), per Table 4 [196]. Typically, neutrophils are recruited by several chemotactic factors released by AT in obesity, primarily IL-8, secreted by inflamed adipocytes. Recruited neutrophils, in turn, release C–X–C motif chemokine ligand 2 (CXCL2), another important neutrophil chemoattractant [71]. Cytosolic constituents and lipids released after adipocyte death are the major signal for the recruitment of neutrophils and macrophages [197]. The latter, by releasing macrophage inflammatory protein 2 alpha (MIP-2α) further attract neutrophils. FFAs released by stressed adipocytes during lipolysis attract and activate neutrophils, stimulating them to produce IL-1β and TNF-α via the NF-kB pathway, which in turn activates other adipocytes and immune cells, promoting local inflammation of AT. IL-1β can activate macrophages in various organs. Moreover, IL-1β, together with TNF-α in the pancreas, can de-differentiate β cells, reducing the expression of the transcription factor FOXO1, which regulates β cell differentiation [71]. Neutrophil elastase disrupts energy expenditure regulation in AT and promotes IR [198]. In hyperglycemia and obesity, neutrophils are activated and release NETs via NADPH oxidase (Nox) activation. There is a causal link between persistent NET formation and MUO in the form of diabetic nephropathy, a complication of T2DM [199]. Increased neutrophil numbers further stimulate excessive macrophage AT infiltration, thereby contributing to IR [166]. In MUO, neutrophils are activated both in circulation and in AT [200], whereas weight loss after BS leads to a reduction in their numbers in both subcutaneous and visceral AT.

Eosinophils. These are present in healthy AT in significant numbers, residing alongside adipocytes and other tissue-resident leukocytes. The stromal–vascular fraction of VAT normally contains ≈5% eosinophils [201]. The recruitment of eosinophils into AT depends on the level of eotaxin CCL11, produced by mesenchymal stem cells and adipocytes in AT [202]. The effect of eosinophils on glucose homeostasis and energy expenditure in AT depends on intercellular interactions within AT. Under physiological conditions, eosinophils, as the main source of local IL-4 in AT, promote the differentiation of preadipocytes into “beige” adipocytes expressing uncoupling protein 1 (UCP-1). This stimulates thermogenesis and increases energy expenditure, thereby restraining obesity [154]. Eosinophil activity in AT is regulated by the transcriptional repressor Krüppel-like factor 3 (KLF3), which controls meteorin-like hormone (METRNL) and IL-33, both of which regulate thermogenesis [203]. Eosinophils produce a range of Th2 cytokines, including IL-4, IL-10, IL-13, and TGF-β. Animal studies have shown that eosinophils play a positive role in inducing M2 macrophages and Th2 cells, and suppress inflammation indirectly via IL-4 and IL-13 [204]. In mouse models, a baseline defect or low number of eosinophils in AT was found to be associated with impaired glucose tolerance, reduced insulin sensitivity, altered adipocyte maturation, increased inflammation in AT, and increased AT mass [205,206]. Caloric restriction in obese mice led to an increase in eosinophil numbers in AT, per Table 4 [207].

Currently, scientific debates are ongoing regarding the role of eosinophils in human obesity induced by a high-calorie diet. Research data indicate that the accumulation of eosinophils in AT may support metabolic health [202]. Obesity-induced metaflammation is associated with a reduction in eosinophil numbers, contributing to the pathological expansion of AT and increased IR risk [208]. It can be hypothesized that, during the transition from MHO to MUO, eosinophil numbers rapidly decline, and impairment of their protective functions further exacerbates the development of chronic inflammation and associated metabolic disturbances.

At the same time, some studies have shown controversial results. Moussa K et al. found that SAT eosinophil content was doubled in patients with metabolic syndrome compared with the control group (*p* = 0.005) [209]. This may be explained by several factors. In particular, changes in subcutaneous fat, which is inherently less metabolically active than visceral fat, may develop more slowly and be less pronounced during the progression of obesity and T2DM compared with the visceral depot. Furthermore, it cannot be ruled out that, at the early stages of MHO to MUO conversion, compensatory mechanisms may be observed, such as a temporary increase in eosinophil numbers followed by a steady decline. In any case, a limited number of studies, and the existing contradictions regarding the role of AT eosinophils in obesity and T2DM require further investigation.

Mast cells. These cells, primarily associated with inflammatory reactions observed in allergies, are found in various AT types. Their precursors are also found in abundance in AT, where, after maturing into mast cells, they interact with adipocytes and participate in immune cell recruitment [210]. Normally, mast cells promote adipocyte differentiation and ensure adequate AT vascularization [211].

Mast cells contain a rich repertoire of pro-inflammatory mediators, including cytokines, chemokines, biogenic amines, proteases (tryptase, chymase), leukotrienes, prostaglandins, etc., and can contribute to AT inflammation [212]. Studies in animal models and humans have shown that, in obesity, the number of mast cells increases in both SAT and VAT [212,213], indicating their potential in metabolic dysregulation. Leptin, which is elevated in obesity, stimulates their activation, degranulation, and cytokine synthesis [214]. The deactivation of mast cells has been shown to promote weight loss, suggesting a potential role for these cells in the regulation of adiposity and energy balance [215]. In AT, mast cells interact with CD8+ T cells and secrete TNF-α, IL-8, and oncostatin [216]. They can also interact with dendritic cells, CD4+ T cells, and Tregs [215,217]. Mast cells promote lipid uptake by macrophages and therefore the formation of foam cells [218]. Their number, particularly in SAT, positively correlates with waist circumference, glucose, triglyceride levels, HOMA-IR, leptin, IL-1β, IL-6, and the activation of p38 MAPK and NF-kB signaling pathways in circulating monocytes, indicating an important role for these cells in the pathogenesis of obesity-associated metabolic syndrome. At the same time, there are some contradictory data regarding mast cells in AT. For example, according to Lopez-Perez et al., patients with MHO had significantly higher numbers of mast cells in AT than patients with MUO [211]. García-Rubio et al. showed that, after BS, the number of mast cells increased 10-fold in VAT and 4-fold in SAT [200,219].

Macrophages. These innate immune cells exhibit high plasticity and easily change their phenotype depending on the microenvironment. Two polar phenotypes, so-called M1 and M2 macrophages, which differ fundamentally in their functions, are known [219,220]. M2 macrophages possess anti-inflammatory potential, characterized by the production of interleukins IL-4, IL-5, IL-10, and IL-13, whereas M1 macrophages are considered pro-inflammatory and produce TNF-α, IL-6, IL-1β, IL-12, and IL-23. The composition and ratio of tissue macrophage types depend on the activating microenvironment stimuli. Accordingly, unsaturated fats and carbohydrates with a low glycemic index promote macrophage polarization towards the M2 phenotype, while saturated fats and simple carbohydrates favor the M1 phenotype [221]. In obesity, the balance between M1 and M2 macrophages undergoes changes. Increased body mass and IR are associated with a shift towards the pro-inflammatory M1 phenotype, along with enhanced migration of monocytes into AT [65,166]. Recruitment of circulating monocytes into AT, which becomes the primary site of inflammation in obesity, is initiated by chemokines (e.g., MCP-1 produced by adipocytes under obesity-associated hypoxia), cytokines secreted by other immune cells (IFNγ from Th1, CD8+ T cells and NK cells), extracellular vesicles from apoptotic adipocytes and neutrophils, and numerous DAMPs generated by uncleared apoptotic cells. Nucleic acids, ATP, mitochondrial DNA, cytochrome c, mitochondrial transcription factor (TFAM), and other nuclear and cytosolic proteins can serve as DAMPs. Secretion of various DAMPs can lead to the production of LTB4, which also attracts monocytes, and participates in a positive feedback amplification loop that sustains chronic inflammation, as seen in diabetes [222]. M1 macrophages are also an important source of ROS and nitric oxide, which can contribute to inflammation and fibrosis in AT. In contrast, resident macrophages in healthy AT are considered metabolically “favorable,” maintaining homeostasis by clearing dead adipocytes and participating in adipogenesis and thermogenesis [223].

It is now recognized that there is a continuum of macrophage subtypes between the M1 and M2 phenotypes. In obesity, both SAT and VAT contain macrophages with a mixed phenotype, so-called metabolically activated macrophages (MMes), which are associated not only with pro-inflammatory function but also with activation of lipid metabolism and expression of ATP-binding cassette subfamily A member 1 (ABCA1), fatty acid translocase ((CD36), involved in fatty acid uptake), and perilipin 2, which regulates lipid droplet formation [224]. Elevated glucose, insulin and palmitates facilitate the polarization of macrophages toward the metabolically activated (MMe) phenotype, highlighting the influence of metabolic factors on macrophage functional status [223,224]. Resident macrophages in unhealthy human AT may have mixed M1/M2 polarization, as they can express M2 markers (CD206 and CD163), while still producing inflammatory cytokines [225]. Another group, CD9+ macrophages, can accumulate lipids and participate in the removal of dead adipocytes via lysosomal exocytosis, which makes them similar to MMe, but they also resemble classical M1/M2 macrophages due to the expression of CD206 and CD11b [226]. CD9+ macrophages may also express the lipid receptor Trem2, which can be activated in response to lysophosphatidylcholine or phosphatidylserine from apoptotic cells [227]. Notably, Trem2+ macrophages prevent adipocyte hypertrophy, inflammation, and metabolic dysfunction [228], which suggests the activation of the reparative function of Trem2+ macrophages in AT in response to inflammation.

Macrophages phagocytosing fragments of dead adipocytes, may initiate the adaptive immune response through antigen capture and presentation to lymphocytes. Notably, due to epigenetic and metabolic changes, macrophages have features of immunological memory, a trait once thought to be unique only to adaptive T and B cells [229,230]. The altered state of macrophages persists and maintains their activation in AT even after weight loss [231]. Toll-like receptor ligands (LPS and IFN-γ) and saturated FFAs (e.g., stearic acid, palmitic acid), found in abundance in obesity, alter macrophage metabolism [232,233].

Macrophages can influence the transition from MHO to MUO. CD95+ macrophages and miR-155 deficiency can correlate with this transition [234]. Additional physiological factors determining the conversion of MHO into MUO include differences in the distribution of M1/M2 macrophage subtypes and differential gene expression in SAT. MHO is characterized by an increased number of M2 macrophages in SAT and is associated with higher expression of genes related to lipid synthesis and transport (fatty acid binding protein 7 (FABP7)), whereas MUO is associated with increased expression of transcripts responsible for inflammatory processes (MCP-1) in AT [235]. The MHO phenotype is also associated with lower activation of the NLPR3 inflammasome and reduced caspase-1 activity in VAT macrophages] [43]. Thus, patients with MHO have a less intense inflammatory profile in AT when compared with those with MUO.

Lymphocytes. CD4+ T helper cells and their subpopulations. Obesity is associated with the accumulation of cytotoxic CD8+ T lymphocytes and CD4+ T helper cells in AT. It is interesting that, according to some reports, activated CD4+ T cells accumulate AT prior to the infiltration by inflammatory macrophages [236,237]. However, other studies demonstrate that it is macrophages and adipocytes in VAT, acting as antigen-presenting cells (APC), that induce the proliferation and differentiation of CD4+ T cells into an inflammatory phenotype. Activation of CD4+ T cells occurs via the cognition by their TCR of an antigen presented by the major histocompatibility complex class II (MHCII) on APC [238], though the key antigens that drive the activation of CD4+ and/or CD8+ T cells remain unclear [158]. In addition, various adipokines (leptin, resistin, TNF-α, and IL-6) regulate the differentiation and function of T lymphocytes. IL-6 promotes the expansion of Th17 cells, which are critical for sustaining inflammation in AT.

The total number of CD4+ T cells and IL-17 production in AT are positively associated with obesity and IR, indicating the development of inflammation. The increase in Th17 cells in AT correlates with HbA1c and glucose levels. The numbers of pro-inflammatory Th1, Th17, and Th22 cells are elevated in both SAT and VAT in patients with MUO compared with those with MHO, whereas anti-inflammatory Th2 and Treg cells help maintain normal AT homeostasis in non-obese individuals and those with MHO, per Table 4 [166]. A higher number of Th2 cells in both VAT and SAT is inversely associated with systemic inflammation and IR in humans [36,239]. Individuals with MUO also exhibit a high proportion of memory CD4+ T cells (CD45RA−CCR7+CD62L+) in AT [240]. Additionally, exhausted CD4+ T cells populations have been described, and their numbers may also increase with the progression of obesity [241,242].

Upon accumulating in AT, T cells alter their phenotype and metabolism, switching to anaerobic glycolysis. Adipokines, cytokines, and lipids from VAT contribute to the formation of new T cell phenotypes, which drives the subsequent progression of inflammation and obesity. Obesity-induced changes in the phenotype of CD4+ T cells lead to dysfunction of the adaptive immune system and persist even after weight loss. This complicates the treatment of obesity-associated metabolic conditions. Obesity facilitates chronic stimulation of CD4+ T cells within AT, resulting in their exhaustion and functional impairment, and manifesting with the expression of inhibitory receptors (PD-1 and CD153) and a distinct transcriptional state [242]. In addition to exhaustion, obesity also accelerates the aging of CD4+ T cells [243]. Aged CD4+ T cells actively secrete osteopontin, which enhances inflammation in VAT [244].

Tregs possessing distinct properties in different depots of AT participate in the regulation of local inflammation and metabolism. Normally, MHCII-mediated antigen presentation determines the development of Tregs in VAT. It is presumed that macrophages and dendritic cells are the main MHCII+ APCs responsible for delivering signals to the TCR of Tregs in visceral fat [245]. Obesity-induced inflammation in AT is accompanied by an imbalance between local pro-inflammatory T cells and Tregs, disruption in metabolism and the impairment in inflammation resolution. As obesity progresses, the number of Tregs in VAT decreases and inversely correlates with BMI and IR. At the same time the Th17/Treg ratio increases [246]. Importantly, obesity increases MHCII expression in adipocytes, and in experiments involving TCR ligation by MHCII expressed on adipocytes, a reduction in Treg numbers in VAT has been observed in obese mice [247]. Similar to findings in mice, visceral fat Tregs in humans play a protective function in obesity and are involved in maintaining glucose levels. However, as obesity develops, adipocytes express higher levels of leptin, which correlates with MHCII expression on adipocytes, and this restrains Treg accumulation in VAT. Interestingly, in individuals with MHO, Foxp3 expression in both SAT and VAT was increased [248].

Cytotoxic CD8+ lymphocytes. Similar to Th1 lymphocytes, CD8+ T lymphocytes demonstrate an increased number in the AT of obese individuals (Table 4), contributing to the recruitment of macrophages [249] and enhancing IL-6 and TNF-α expression by adipocytes [65]. CD8+/CD4+ ratio is also increased in obesity. A comparative study found no significant differences in the relative proportions of CD8+ T cells between MHO and MUO groups; however, the absolute number of CD8+ cytotoxic T cells was higher in individuals with MUO [240].

Obesity promotes the formation of pathological CD8+ T cells, whose activation occur in response to endogenous AT antigens presented by MHC class I, or exogenous antigens derived from food or released by the gut microbiome [250,251]. Adipocytes can activate CD8+ T cells in individuals with obesity. The infiltrating CD8+ T cells have a restricted TCR-Vβ repertoire, which narrows the range of recognized antigenic epitopes but enhances the specificity of binding to antigens within the inflamed AT [251]. Antigen-specific activated cytotoxic CD8+ T cells, accumulating in VAT, can potentially secrete large amounts of IFNγ, recruit monocytes/macrophages, and destroy hypertrophied adipocytes. Notably, obesity may promote lipid uptake by activated cytotoxic CD8+ cells, shifting their metabolism from glycolysis towards fatty acid oxidation [252].

The role of CD8+ T lymphocytes in AT inflammation is not fully understood. It has been shown that CD8+ T lymphocytes are heterogeneous and include two subpopulations: Tc1 cells, characterized by hyperproduction of IFNγ, IL-12, and IL-18, and Tc2 cells, which produce anti-inflammatory cytokines IL-4 and IL-5. It is possible that leptin can shift the differentiation of CD8+ cytotoxic T cells towards the Tc1 phenotype and, via the STAT3-dependent signaling pathway, enhance PD-1 expression, leading to cytotoxic T cell exhaustion [252].

B lymphocytes. In healthy AT B cells maintain homeostasis by supporting an anti-inflammatory microenvironment, primarily through the secretion of IL-10 and IgM [186]. Obesity is accompanied by the trafficking of B cells into VAT, their accumulation, and a shift in their subpopulation balance and functional activity towards a pro-inflammatory state. By modulating T cell responses and producing pathogenic antibodies under an altered obese microenvironment, B cells contribute to metaflammation and further metabolic dysfunction of AT, including the development of IR.

As obesity progresses, the composition of B cell subpopulations changes. The frequency of pro-inflammatory IgD-CD27- DN B cells, which secrete pathogenic autoantibodies and increase in the periphery during obesity, also rises in AT. Conversely, the proportion of CD20+CD27+CD43+ B cells and IgM antibodies, which inhibit the development of inflammation and IR, decreases in both SAT and VAT during obesity and metabolic syndrome [253]. The proportion of Bregs, which maintain the homeostasis of healthy AT and exert anti-inflammatory effects through IL-10 secretion, is also reduced in obesity compared with healthy AT [186]. Normally, IL-10 produced by B cells promotes polarization of macrophages towards the M2 phenotype. In humans, obesity has been shown to be associated with a reduction in CD27+CD38high memory B cells, as well as in IL-10-secreting Bregs [130].

Numerous mechanisms have been proposed to explain the pathogenic involvement of B cells in AT dysfunction. Adoptive transfer of B2 cells into the VAT of mice promoted the development of metabolic disturbances, as these cells likely produce pro-inflammatory cytokines and contribute to IR through the secretion of LTB4 [254], as well as by activating macrophages and suppressing Tregs [36]. In obesity, B cells, acting as antigen-presenting cells, can present antigens to T cells (Th1 and CD8+ Tc), thereby enhancing IFNγ production and promoting macrophage polarization towards the M1 phenotype. B cells also undergo phenotypic changes, switching antibody classes and beginning to produce IgG (including IgG2c), which enhances TNFα secretion by macrophages [255]. Additionally, B cells secrete chemokines (such as IL-8), which attract new immune cells into AT, forming a positive feedback loop that sustains inflammation. Furthermore, T-bet+ B cells, able to secrete CXCL10 and which can induce pancreatic β-cell dysfunction, are also found in obese AT [256,257].

Natural Killer Cells. Like other cells of the innate immune system, NK cells play an important role in the early stages of inflammation development in AT. Moreover, they play the role of sensors of AT metabolic stress [258]. Accumulation of NK cells in VAT is observed in humans with obesity [132]. In addition, mice models support the idea that a reduction in NK cell numbers in fat is associated with improved insulin sensitivity and a decrease in total macrophage numbers [259]. IL-12 and IL-15, which appear in AT at the onset of obesity, lead to local accumulation of NK cells, primarily through proliferation rather than recruitment from the circulation. When analyzing NK cell subsets we find that there is a notable 2.5-fold increase, specifically in the CD56brightCD16- NK cell population, in AT during obesity [259]. By producing excessive amounts of IFNγ and TNF-α in AT [260], these cells promote the polarization of macrophages towards the M1 phenotype and the development of IR [189,259]. In mice on a high-fat diet, the number of NK cells in VAT increases three- to five-fold, and stressed adipocytes show increased expression of the ligand for NKp46, one of the activating receptors of NK cells, thereby stimulating NK cells to produce IFNγ [189]. Mice deficient in NK and NKp46 developed obesity on a high-fat diet but did not develop IR, highlighting the critical role of NK cells in metaflammation and the development of MUO. Thus, despite many stress factors contributing to the macrophage accumulation and inflammation in AT, the surpassing of a certain threshold of NKp46 ligand expression on adipocytes may be the key point at which NK cells provide the immunological “license” for macrophages to switch to the pro-inflammatory M1 phenotype and subsequently drive IR [261].

Innate Lymphoid Cells (ILCs). Being tissue-resident lymphocytes, ILCs comprise a heterogeneous group of lymphoid lineage cells which lack antigen-specific B or T cell receptors. ILCs include NK cells and helper ILCs of three subtypes (ILC1, ILC2, and ILC3). Obesity and metabolic syndrome are associated with the accumulation of ILC1 in SAT and VAT, accompanied by excess IFNγ and M1 polarization of macrophages [262]. In contrast, ILC2 are more commonly found in AT in the absence of obesity and inflammation, where they help maintain eosinophil and M2 macrophage activity; their numbers decrease in obesity and metabolic syndrome [263].

Comparing the AT cellular composition under different metabolic status is a complex task, as most available data have been obtained from mouse models, with analysis of various visceral fat depots (epididymal, perigonadal fat, etc.), whereas data on human AT are quite limited and mainly pertain to subcutaneous fat, partly due to technical limitations in obtaining tissue samples (Table 4). These circumstances may explain some of the contradictions in current findings regarding the dynamics of AT cellular composition in MHO and MUO.

**Table 4:** Changes of adipose tissue immune cells in healthy, MHO and MUO both in human and mice.

Cell Type	Healthy	MHO	MUO	Type of Study	References
**Pre-adipocytes, % of SVF (mean ± SE)**	82.6 ± 2.9	74.4 ± 2.3	65.6 ± 2.9 (# lean vs. MUO and MHO vs. MUO)	Cross-sectional	[194]
Granulocytes, % of SVF (mean ± SE)	3.8 ± 1.1	6.4 ± 1.6	13.0 ± 2.2 (# lean vs. MUO and MHO vs. MUO)	Cross-sectional	[194]
*Neutrophils, % of SVF in mice with obesity in epididymal fat*	*~0.1*	*Up to 1.5, (#)*	*In mice with active ELANE gene (neutrophil elastase), tissue sensitivity to insulin was worse (20% suppression of gluconeogenesis in the liver compared with 60% in NE knockout mice, #)*	*Cross-sectional*	[196]
*Neutrophils, % of all immune cells in AT of mice*	*~1.0*	*10–20-fold increase in the number of neutrophils in visceral AT of mice*	*NA*	*Cross-sectional*	[71,264]
*Macrophages, F4/80+ cells, % of immune cells in AT of mice, mean ± SD*	*12 ± 2 in SAT and VAT*	*41 ± 4 (##, in SAT and VAT)*	*NA*	*Cross-sectional*	[139]
Macrophages, % of immune cells in human AT	~10–15 in SAT (?)	Up to 40–60 in SAT (?), metabolic status not specified	NA	Cross-sectional	[265]
Macrophages, % of the total number of adipocytes in humans, mean ± SEM	NA	4.1 ± 1.2 in SAT and 6.3 ± 1.7 in VAT	9.1 ± 1.9 in SAT (# for MUO and MHO) 14.6 ± 4.1 in VAT, (NS for MUO and MHO)	Cross-sectional	[213]
**Macrophages, % of SVF (mean ± SE)**	6.1 ± 1.1	7.9 ± 0.7	10.8 ± 1.2 (# lean vs. MUO and MHO vs. MUO)	Cross-sectional	[194]
**NLRP3 expression in human VAT, means, data were normalized according to HPRT1 mRNA levels**	~0.5	~1	~3 (# lean vs. MUO and MHO vs. MUO)	Cross-sectional	[194]
Eosinophils, % of SVF, mean ± SEM/×10^3^ cells per 1 g of VAT in mice, mean ± SEM	~6.4 ± 0.9% or ~25 ± 5 × 10^3^ cells per 1 g	~1.1 ± 0.3% (*) or 11.6 ± 4 × 10^3^ cells per 1 g (#)	NA	Cross-sectional	[205]
**Mast cells, ×10^5^ cells/mm^2^ in human AT, mean ± SEM**	~1.6 ± 0.45 × 10^5^ cells/mm^2^ of mast cells expressing chymase in VAT	~0.5 ± 0.23 × 10^5^ cells/mm^2^ of mast cells expressing chymase in VAT, NS	The number of two mast cell phenotypes (expressing chymase and tryptase) was significantly increased (max 10 ± 5.9 × 10^5^ cells/mm^2^) in both fat depots (# for MUO vs. MHO)	Cross-sectional	[213]
**Mast cells, % Astra Blue positive/expressing tryptase in human SAT, median (IQR)**	NA	~20 (10–20.5) (Astra Blue positive); ~40 (10–50) (expressing tryptase)	~50 (40–60) *** (Astra Blue positive), ~60 (40–80) (expressing tryptase) *	Cross-sectional	[212]
NK cells, cells*10^3^ in VAT of mice, mean ± SEM	~3.1 ± 0.7 * 10^3^	~6.8 ± 1.6 * 10^3^ (#)	*NA*	*Cross-sectional*	[189]
NK cells in human VAT, % of live cells, Me [IQR]	2.7 [1.64–4.09]	~3.79 [2.66–6.02], (# for MHO+MUO versus healthy)	NA	Cross-sectional	[132]
*CD8+ lymphocytes, % of TCRαβ+ cells in epididymal fat of mice, mean ± SEM*	*5 ± 1.1*	*16 ± 1.1 (#)*	*NA*	*Cross-sectional*	[266]
CD8+ lymphocytes, % of total T cells in human VAT, mean ± SD	NA	38.4 ± 6.8	NA	Cross-sectional	[239]
CD8+ lymphocytes, % of CD3+ cells in human VAT, mean ± SEM	~40 ± 2.5	~45 ± 2.5, NS	NA	Cross-sectional	[240]
Th1 lymphocytes, % of CD4+ cells or n cells g^−1^*10^3^ in mice, mean ± SEM	41.3% or ~20.0 ± 4.0 cells·g^−1^*10^3^ in VAT; 14.9% or 10 ± 0.5 cells g^−1^*10^3^ in SAT	50.3% or ~60 ± 14 cells·g^−1^*10^3^ in VAT, (#); 32.4% or 38 ± 2.0 cells g^−1^*10^3^ in SAT, (#)	*NA*	*Cross-sectional*	[267]
Th1 lymphocytes (CD4+ IFN-γ), % of CD4+ cells in human SAT, mean ± SD	57 ± 24	66 ± 22	68 ± 13, NS for all groups.	Cross-sectional	[268]
Th2 lymphocytes (GATA3+ cells), % of CD4+ cells or n cells·g^−1^*10^3^ in VAT of mice, mean ± SEM	33.7% or 14.9 ± 0.5 cells·g^−1^*10^3^	15.3% (#) or 19 ± 4.5 cells·g^−1^*10^3^, NS	*NA*	*Cross-sectional*	[267]
Th2 lymphocytes (CD4+ IL-13), % of CD4+ cells in human SAT, mean ± SD	13.0 ± 9.7	14.1 ± 10.2	13.0 ± 9.7, NS for all groups	Cross-sectional	[268]
Th17, % of CD4+ cells or n cells·g^−1^*10^3^ in mice, mean ± SEM	1.97% or 1.1 ± 0.5 cells·g^−1^10^3^ in VAT; 2.1% or 1.2 ± 0.06 cells·g^−1^10^3^ in SAT	0.58% or 0.8 ± 0.125 cells·g^−1^10^3^ in VAT and NS; 2.93% or 2.8 ± 0.1 cells·g^−1^10^3^ in SAT (#)	NA	Cross-sectional	[267]
**Th17 (CD4+ IL-17), % of CD4+ cells in human SAT, Me [Q1; Q3]**	~1.2 [0.5; 4.0]	~1.8 [0.5; 5.0]	~14 [2.8; 17], (# for MUO versus MHO and healthy)	Cross-sectional	[268]
**Th22 (CD4+ IL-22), % of CD4+ cells in human SAT, Me [Q1; Q3]**	~2 [0,5; 11]	~4 [1,2; 6,8]	~11 [4.5; 18], (# for MUO versus MHO and healthy)	Cross-sectional	[268]
Treg lymphocytes, % of CD4+ cells or n cells·g^−1^*10^3^ in VAT of mice, mean ± SEM	29.0% or 13.6 ± 1.4 cells·g^−1^*10^3^	7.9% (#); or 10.5 ± 0.2 cells·g^−1^*10^3^, NS	*NA*	*Cross-sectional*	[267]
**Tregs, means, % among the CD4+ T cell population from the human VAT**	~5	~3	~2.5 (# lean vs. MUO and MHO vs. MUO)	Cross-sectional	[194]
Treg lymphocytes, % of CD4+ T cells in human AT, mean ± SEM	19 ± 3.6 in VAT; 24 ± 4.5 in SAT	3.5 ± 4.3 in VAT and 7 ± 2.9 in SAT, (*** for MHO + MUO vs. healthy)	NA	Cross-sectional	[269]
B lymphocytes in VAT of mice, % of SVF	~10	~20 (#)	NA	Cross-sectional	[254]
B lymphocytes, % of CD45+ cells in SVF in gonadal adipose tissue of mice, mean ± SD	~2 ± 1	~5.5 ± 3.5 (*)	NA	Cross-sectional	[270]
CD19+CD27+CD38^high^ memory B cells, cells/mm^3^ in human SAT, mean ± SD	2.8 ± 1.7	0.3 ± 0.2 (#), metabolic status not specified)	NA	Cross-sectional	[130]
**Innate lymphoid cells (ILCs type 1), cells mg^−1^ in human omentum, mean ± SD**	5 ± 3	15 ± 3	23 ± 3 (** for all groups)	Cross-sectional	[262]
**IL-1β expression in human VAT, means, data were normalized according to HPRT1 mRNA levels**	~1	~4	~10 (** lean vs. MUO, ** MHO vs. MUO)	Cross-sectional	[194]

Notes to Table 4. *—*p* < 0.01; **—*p* < 0.001; ***—*p* < 0.0001; #—*p* < 0.05; ##—*p* < 0.005, NS—non significant. «NA» Data are not available. SVF—stromal–vascular fraction. Mice data are presented in italics. Data marked in bold are potential predicting MUO biomarkers.

Thus, it can be concluded that AT dysfunction, with its structural remodeling, pro-inflammatory cellular infiltration, and production of a wide range of pro-inflammatory cytokines observed in obesity and metabolic syndrome, leads to the loss of normal insulin sensitivity in AT and the subsequent development of prediabetes/T2DM. The transition from MHO to MUO is accompanied by an increase in the numbers of neutrophils, macrophages (M1), mast cells, B cells (IgD-CD27- DN, T-bet+), pro-inflammatory Th1, Th17, Th22, CD8+ T cells (with an increased Tc1/Tc2 ratio), as well as memory CD4+ T cells with the CD45RA-CCR7+CD62L+ phenotype and exhausted CD4+ cells, CD56^bright^CD16- NK cells, and ILC1, along with a simultaneous decrease in M2 macrophages, eosinophils, Tregs, Th2, Bregs, CD20+CD27+CD43+ B cells, and CD19-CD27+CD38^high^ memory B cells. Although many studies show similar trends in dynamical changes for immune cell subpopulations, their use as predictive biomarker of the MHO/MUO transition requires further longitudinal investigation with determination cutoff points and validation.

**Figure 3:**
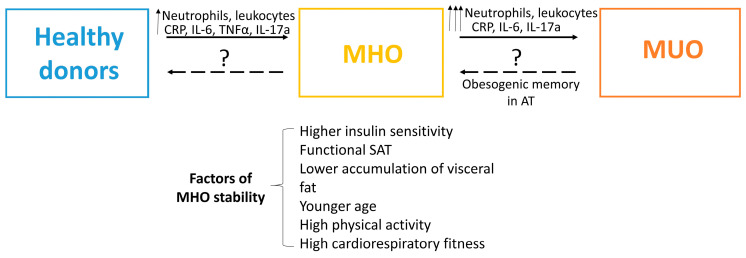
**Hypothetical model of interrelations between healthy state, metabolically healthy obesity (MHO) and metabolically unhealthy obesity (MUO).** Such pro-inflammatory mediators as CRP, IL-6, IL-17a and circulating cells (total leukocytes and neutrophils), and their dynamic increase can serve as potential biomarkers of MHO/MUO transition. Horizontal arrows denote the obesity progression from a healthy phenotype to a metabolically unhealthy phenotype, concomitant with the loss of protective factors. Small vertical arrows signify an escalation of inflammation. Dashed arrows represent potential MUO/MHO transition as well as return to the healthy state from MHO level. A question mark highlights as yet unidentified the protective ratio between anti-inflammatory and pro-inflammatory mediators that may underpin the interconnection between the healthy phenotype and the MHO/MUO phenotypes.

Based upon our analytical review we conclude that, at present, though some parameters demonstrate significant differences in multiple cross-sectional studies, only a few of them (CRP, IL-6, IL-17A, and absolute counts of leukocytes and neutrophils) meet the criteria of validated biomarker (Figure 3).

## 7. Reversibility of Metaflammation Following T2DM Remission Bariatric Surgery (BS) and After Incretin Receptor Agonists Therapy: Metabolic Memory

At present, bariatric surgery (BS) and pharmacological treatment with GLP-1 and GIP receptor agonists are considered among the most effective strategies for managing obesity and T2DM. The advent of BS marked a turning point in the treatment of obesity and T2DM, as it appeared to be not only highly effective in fighting against excess weight but also introduced the concept of diabetes remission. T2DM remission is defined as achieving an HbA1c level of ≤6.5% for at least three months in the absence of glucose-lowering therapy. Due to its potential to achieve T2DM remission, BS is also recognized as “metabolic” surgery [271].

BS can be divided into two main groups based on surgical technique: restrictive procedures, which primarily limit food intake (gastric banding, intragastric balloon, sleeve gastrectomy), and combined procedures, which incorporate both restrictive and malabsorptive components (Roux-en-Y gastric bypass, one-anastomosis gastric bypass/mini gastric bypass (OAGB-MGB), biliopancreatic diversion with SADI and duodenal switch modifications). Currently, the most performed procedures are sleeve gastrectomy (SG), gastric bypass (GB), and biliopancreatic diversion (BPD). Weight loss after BS is often assessed in terms of excess weight loss percentage (EWL). According to a systematic review by L. Fischer et al., EWL for SG averages 56.1% and 68.3% for GB [272], while, according to P.E. O’Brien et al., EWL for BPD reaches 74.1% or more [273], making BPD the most effective intervention for both weight loss and T2DM remission.

It has been shown that, during the first year after BS, both SAT and VAT decrease in volume [274], with a reduction in the size of subcutaneous [275] and visceral adipocytes [276,277]. Human studies have shown that the size of subcutaneous adipocytes decreases after BS, resembling those in non-obese individuals [60,278]. Data on changes in the size and number of visceral adipocytes after BS are limited due to rare postoperative VAT sample collection.

After BS, most patients experience rapid weight loss and marked improvements in lipid and glucose metabolism. This raises the important questions: does BS lead to a resolution of the pathological inflammatory characteristic of MUO in patients with T2DM? Is it possible to discuss the reversibility of MUO and its conversion to MHO or to a completely “healthy” phenotype? Indeed, according to several studies, BS can shift the adipokine balance towards an anti-inflammatory profile [279] and even reverse AT dysfunction and inflammation, regardless of EWL after BS [280]. Recent research shows that, in the long term, circulating adiponectin levels increase significantly (by about 12%), while leptin levels decrease (by about 45%) after BS [281,282]. Similar results have been shown for reduction in other pro-inflammatory cytokines and adipokines (TNF-α, resistin) [282,283,284] and increase in anti-inflammatory adipokines (omentin) [285], collectively indicating a regression of inflammatory changes in AT after BS, highlighting the modulatory effect of BS on adipose tissue inflammation and structure. BS affects the systemic inflammatory status of patients by increasing the level of anti-inflammatory cells producing IL-10, including B lymphocytes, while reducing pro-inflammatory cells producing IL-6, as shown three months after GB [286]. Twelve months after SG, a shift from a pro-inflammatory to an anti-inflammatory pattern was also observed, accompanied by a 25% decrease in the percentage of CD45RO+CD27-CD4+ effector memory T cells (26.38 ± 9.49 after BS vs. 34.63 ± 9.24 before BS) and a 50% increase in circulating Tregs (3.2 ± 0.54 after BS vs. 2.08 ± 0.34 before BS), although not reaching levels of Tregs seen in healthy individuals (7.07 ± 0.5) [281]. At the same time, some studies demonstrate that, despite significant weight loss and improved metabolic status after BS, the altered pro-inflammatory cytokine profile persists in patients with obesity and T2DM (patients with T2DM after surgery had elevated levels of IFN-β, IL-27, IL-1α, IL-2, regenerating islet-derived protein 3A, visfatin, osteopontin [287], serum amyloid A, and MCP-1 [288]).

Numerous studies report a reduction of inflammation in both SAT and VAT after weight loss resulting from BS [60,166,200,289,290]. In the study by V.A. Palomäki et al., the dynamics of SAT macrophage infiltration was examined before and 12 months after BS (60 patients with morbid obesity, 62 controls). The number and density of “crown-like structures” formed by macrophages around apoptotic adipocytes decreased from 4.1 to 1.1 per 1000 adipocytes after surgery [289]. Furthermore, several studies have demonstrated that there was also a shift towards a predominance of anti-inflammatory macrophages in both SAT and VAT after BS [166,290]. Weight loss after BS in humans was also found to be accompanied by a reduction in neutrophil numbers in both fat depots, whereas no significant changes in T cell levels were observed [200]. An interesting finding was that, after BS, the number of mast cells increased in both SAT and VAT, as did the number of adipocyte progenitor cells, which was associated with a favourable immunophenotypic profile of AT [200].

At the same time, several studies have found no positive changes in AT after weight loss due to BS [291,292,293]. Despite marked metabolic improvements observed with weight loss, persistent inflammatory changes in visceral fat may remain both in humans after BS and mice under caloric restriction [294,295], characterized by continued pro-inflammatory macrophage polarization and no change in Treg cell numbers [292,293,296]. D.K. Hagman et al. showed that CRP levels decreased over 12 months of follow-up after BS in humans, while adiponectin levels increased. However, there was no regression of inflammation in SAT, while the number of neutrophils increased by 15–20 times (141,006 (92,300–194,407) vs. 12,019 (5845–21,287) cells per g of SAT, *p* < 0.001), without significant changes in the amount of other leukocyte subsets [291]. Thus, several studies support the hypothesis of “metabolic memory” following weight loss [297,298]. Indeed, given the gradual development of inflammation in obesity, many researchers anticipated a resolution of inflammation after weight loss [158]. Unexpectedly, however, inflammation may persist following weight loss, both in the periphery and in AT [158]. The activity of CD4+ and CD8+ T cells producing type 1 and type 17 cytokines is maintained, driven by macrophages that remain in AT. Thus, despite the restoration of carbohydrate metabolism, improved insulin sensitivity, and reduced level in cholesterol and triglycerides, AT maintains a metabolic memory by preserving the inflammatory microenvironment established earlier during obesity. Indeed, BS cannot instantaneously alter the structure of AT, as the cellular composition cannot be renewed immediately.

The close relationship between immunity and metabolism, as well as the phenomenon of worsening metabolic health (characterized by reduced glucose tolerance and insulin sensitivity) during multiple weight cycling episodes suggest a hypothesis that metabolic changes may lead to the formation of immunological memory, which is retained by the immune system [243,299]. The immune system is indeed capable of retaining memory of all antigens it has encountered. Information regarding metabolic status may be “memorized” by both adaptive immune cells (long-lived memory T cells) and trained cells of innate immunity. For instance, macrophages possess long-term memory for specific antigens and respond with greater intensity upon re-exposure to a previously encountered obesogenic stimulus. Epigenetic (such as histone modifications, DNA methylation) and metabolic changes in macrophages (e.g., glycolysis) determine the immune memory responses of macrophages [229,230]. Immune memory responses during weight loss can enhance the inflammatory response and maintain macrophages in an activated state, thereby promoting weight regain [231]. Notably, unlike adaptive immunity, macrophages are less selective; they may respond to the secondary stimulus while not precisely matching the primary one, or they may even be of a different nature, not associated with obesity but, for example, with hypertension [300].

Among adaptive immune cells, CD4+ and CD8+ effector memory T cells (Tem) in AT have been identified as critical in forming obesogenic memory, which contributes to weight regain during weight fluctuations. A specific role of Th1 and Th17 cells growing in number during weight regain after weight loss has been noted [158]. The presence of CD4+ and CD8+ Tem cells in AT, as well as a reduced T-cell receptor repertoire, supports the existence of obesogenic antigens that stimulate T cells and maintain the memory cell pool [179]. Although macrophages, dendritic cells, B cells, and adipocytes are potentially capable of presenting antigen to T cells in AT, it currently remains unclear what type of antigen-presenting cell is involved and what specific antigen participate in T-cell activation in obesity [299]. Prolonged stimulation of T-cell receptors by antigen, as observed in chronic inflammation in obesity, may lead to the emergence of both functionally exhausted and senescent immune cells, which differ in both phenotype and functional characteristics [301]. A key distinguishing feature of senescent cells, as opposed to exhausted cells, is their increased capacity to produce suppressive and pro-inflammatory cytokines (TGF-β, IL-10, as well as TNF-α, IL-8, IL-6, IL-2, IFN-γ), contributing to the unique senescence-associated secretory phenotype (SASP) associated with T2DM [168]. Notably, senescent CD4+ and CD8+ T cells with the CD45RA+CD28-CD27-CCR7-KLRG1high phenotype are elevated in T2DM [170,171]. The accumulation of senescent cells negatively affects the immune system. Furthermore, in addition to immune cells, the persistence of the transcriptional and functional memory of obesity has also been reported in adipocytes and endothelial cells [302].

Thus, an individual patient’s “immunological content” is formed, which does not disappear following BS: upon encountering an old stimulus, cells retaining memory of the obesogenic antigen respond with an intense inflammatory reaction. Moreover, the recurrence of T2DM after BS may also be explained by the patient’s obesogenic memory. The persistence of cellular memory warrants consideration, as proinflammatory cell activity may sustain ongoing metaflammation in obesity. Previously, predictors of T2DM relapse after BS included indicators of a more severe preceding course of T2DM: longer disease duration, higher HbA1c levels, lower C-peptide and insulin levels, and a greater number of glucose-lowering medications. The return of diabetes was also found to be associated with a more severe and prolonged course of T2DM prior to BS and an “exhaustion” of pancreatic reserves. Currently, it is hypothesized that a more severe course of T2DM before BS is accompanied by more pronounced and persistent inflammation of AT, which does not resolve after BS, thereby predisposing to T2DM relapse [293,303].

Use of both GLP-1 receptor agonists and dual GLP-1/GIP receptor agonists has recently gained increasing attention in clinical settings [304]. This circumstances also raises an important inquiry: what impact do these pharmacological agents have on metaflammation and the functional characteristics of AT in individuals with obesity and T2DM?

It has been established that GLP-1 receptors are expressed on various immune cell types, suggesting a biological basis for their potential direct role in modulating inflammatory responses [305]. Activation of these receptors triggers pathways mediated by AMP-activated protein kinase (AMPK), leading to suppression of nuclear factor kappa-light-chain-enhancer of activated B cells (NF-κB) signaling and promoting the induction of regulatory T cells via Foxp3. As a result, levels of pro-inflammatory cytokines such as TNF-α and IL-6 are decreased [306]. Moreover, GLP-1-based therapies inhibit the infiltration of cytotoxic T cells into tissues, while also reducing neutrophil adhesion and activity within the endothelium [307]. These agents also induce a shift in macrophage polarization from M1 to M2 phenotype, contributing to a reduction in general inflammatory markers such as ROS, COX-2, and CRP [306]. Such anti-inflammatory effects are reciprocally linked to improvements in metabolic disturbances like insulin resistance [306].

Emerging evidence indicates that GLP-1 receptor agonists decrease the prevalence of M1 macrophages within VAT and encourage a transition toward M2-like macrophages. This immunological shift may lead to lower cytokine concentrations that otherwise inhibit the activity of key thermogenic regulators, including PGC-1α and PRDM16 [308]. By attenuating inflammatory signaling, GLP-1RAs appear to improve adipocyte plasticity, stimulate mitochondrial biogenesis, enhance oxidative metabolism, promote thermogenesis, and ultimately boost whole-body energy expenditure [309]. In addition to macrophage polarization, the immunomodulatory effects of GLP-1RAs seem to involve other immune cell populations, such as Treg cells, eosinophils, and ILC2s, which contribute to the browning of white adipose tissue and the development of beige and brown adipocytes [310]. It remains unclear how long the mentioned effects of incretin receptor agonists persist after discontinuation or interruption of therapy.

## 8. Strengths and Limits

The review provides a comprehensive overview of the immunological landscape of MHO and MUO based on a detailed comparative analysis of a large number of circulating mediators and immune cells of blood and adipose tissue. However, at present only a small set of parameters (CRP, IL-6, IL-17A, and absolute leukocyte and neutrophil counts) might serve as prognostic biomarkers (AUC up to 0.6–0.86). An important strength of the work is the examination of the effects of bariatric surgery and incretin receptor agonists on metaflammation, considering the phenomenon of ‘metabolic memory’. Nevertheless, these benefits are offset by serious limitations. The review is classified as a narrative review. A key challenge in the field is the absence of universally accepted criteria for defining MHO and MUO. Data derived from human adipose tissue, particularly from the visceral depots, remain limited owing to technical and ethical challenges. Changes in the liver, skeletal muscles and pancreas are described in less detail, despite their important role in systemic insulin resistance.

## 9. Conclusions

It has been established that sex (with a higher rate of MHO/MUO transition in men), age, and physical activity substantially modify the inflammatory profile. For most immunological parameters (e.g., the percentage of regulatory T cells, T helper 17 cells, the neutrophil-to-lymphocyte ratio, and others), only tentative cut-off values have been proposed that have not undergone independent validation in different populations. Currently, only a small number of pro-inflammatory molecules and cells might be classified as promising biomarkers for categorizing obesity phenotypes. Validation of their effectiveness as biomarkers requires standardized guidelines and extended longitudinal studies. The concept of ‘metabolic memory’ is becoming important for preventing type 2 diabetes recurrence and improving the efficacy of obesity therapies.

## Figures and Tables

**Table 1:** General characteristics of metabolically healthy obesity and metabolically unhealthy obesity.

Metabolically Healthy Obesity	Metabolically Unhealthy Obesity
Younger age, mean [SD] 37.7 [10.7] [9]	Older age, mean [SD] 42.9 [10.7], *p* < 0.001 [9]
Normal blood pressure, moderate prevalence of atherosclerotic cardiovascular diseases and other associated conditions [9]	Arterial hypertension, high prevalence of atherosclerotic cardiovascular diseases [9]
Elevated levels of cell adhesion molecules (sICAM-1), E-selectin, P-selectin [53], initial endothelial changes [54]	Significantly increased levels of cell adhesion molecules [53], increased levels of AGEs, endothelial dysfunction [54]
Close to normal lipid profile [9], maintenance of relative balance between lipolysis and lipogenesis [7]	Dyslipidemia [9], enhanced lipolysis and release of FFAs [7]
Maintenance of relative insulin sensitivity	Insulin resistance, hyperglycemia, T2DM [9]
Preservation of main liver functions [9]	Metabolic- associated fatty liver disease (MAFLD) [9]
Predominant subcutaneous fat deposition, fat on lower extremities, gluteal region, thighs [9]	Excessive fat deposition in the liver or visceral depot [9]
Maintenance of normal muscle function [47]	Myosteatosis, muscle tissue inflammation, oxidative stress [47]
Less prominent metaflammation than in MUO (increased levels of CRP, IL-6, TNF-α), but more profound than in healthy individuals [13] Higher levels of anti-inflammatory cytokines compared with MUO (adiponectin, IL-10) [54,55]	Distinct elevation of metaflammation markers (IL-6, IL-8, TNF-α, MCP-1, CRP, leptin, resistin, PAI1) [13,35,36]
Less prominent increase in circulating leukocytes, neutrophils, and monocytes than in MUO; balance of Th1/Th2 and Th17/Treg ratios; relatively preserved pool of Tregs and Bregs [14,56,57,58]	Significant increase in circulating leukocytes, neutrophils, monocytes, B cells, and CD4+ T cells; increased Th1/Th2 and Th17/Treg ratios; decreased proportion of Tregs and Bregs [14,56,57,58]
Preservation of adequate adipogenesis, with hyperplasia predominating over adipocyte hypertrophy; hypoxia, adipocyte apoptosis, and AT fibrosis are less prominent than in MUO [59,60] Adipocyte sizes are smaller than in MUO [61]	Significant pathological changes in AT: adipocyte hypertrophy, impaired angiogenesis, hypoxia, adipocyte apoptosis, AT fibrosis [59,60]
Pro-inflammatory cell infiltration in AT is more pronounced than in healthy individuals but less than in MUO; higher number of anti-inflammatory cells compared with MUO [61,62]	Increased secretion of pro-inflammatory cytokines in AT, marked inflammatory cell infiltration in AT [35,36,62]

Notes to Table 1. MUO—metabolically unhealthy obesity; CRP—C-reactive protein; IL-6—interleukin-6; TNF-α—tumor necrosis factor α; IL-10—interleukin-10; Th1—T helper cell type 1; Th2—T helper cell type 2; Th17—T helper cell type 17; Tregs—regulatory T cells; Bregs—regulatory B cells; AT—adipose tissue; IL-8—interleukin-8; MCP-1—monocyte chemoattractant protein-1; PAI1—plasminogen activator inhibitor-1; T2DM—type 2 diabetes mellitus; FFAs—free fatty acids; AGEs—advanced glycation end-products.

**Table 2:** Pro-inflammatory factors in the peripheral blood of healthy individuals and patients with MHO and MUO.

Parameters	Healthy Individuals	MHO	MUO	Type of Study, Biomarker Validation	References
Free fatty acids, mmol/L, mean ± SD	0.22 ± 0.21	0.49 ± 0.19 (* for MHO and healthy)	0.55 ± 0.27, (* for MUO and healthy; MUO and MHO)	Cross-sectional	[53]
**Oleic acid/stearic acid—OA/SA (C18:1 n9/C18:0), mmol/L, mean ± SD**	0.65 ± 0.31	0.56 ± 0.23 (# for MHO vs. healthy)	0.85 ± 0.30 (* for MUO vs. MHO)	Longitudinal, AUC = 0.755~0.767 for differentiation between MHO and MUO	[73]
AGEs, AU, mean ± SD	1.81 ± 0.22	1.86 ± 0.51	2.44 ± 0.67, (** for MUO and healthy; MUO and MHO)	Cross-sectional	[74]
**CRP, mg/L, mean ± SD**	3.0 ± 5.2	5.7 ± 6.7	6.0 ± 6.5, (** for all groups.)	Cross-sectional	[12]
	Longitudinal, AUC = 0.69 (***) for CRP in predicting MUO				[75]
	Longitudinal, AUC = 0.61 (95% CI: 0.59–0.63) (*) in predicting MUO				[76]
	CRP levels in MHO are higher than in healthy lean		CRP levels in MHO are lower than in MUO	Meta-analysis	[7]
**IL-6, pg/mL, mean ± SD**	1.3 ± 1.1	1.8 ± 1.1	1.9 ± 1.3, (** for all groups)	Cross-sectional	[12]
	1.9 ± 0.4 (# vs. MHO and ** vs. MUO)	2.3 ± 0.8 (# vs. MUO)	2.6 ± 1.0	Cross-sectional, AUC = 0.67 for differentiation between MUO vs. MHO, cut-off = 2.02 pg/mL	[77]
	Longitudinal, AUC = 0.86 (***) for IL-6 in predicting MUO				[75]
	IL-6 levels in MHO are higher than in healthy lean		IL-6 levels in MHO are lower than in MUO	Meta-analysis	[7]
TNF-α, pg/mL, mean (95% CI)	10.70 (9.76–11.64)	11.41 (9.85–12.96)	11.43 (8.73–14.14), NS for all groups	Cross-sectional	[55]
TNF-α, ng/L, mean	Cross-sectional, AUC = 0.765, cut-off = 93.4 in predicting MUO				[78]
	TNF-α levels in MHO are higher than in healthy lean		NS for MUO vs. MHO	Meta-analysis	[7]
IFN-γ, pg/mL, mean ± SEM	402.4 ± 14.3	446.1 ± 17.8	434.6 ± 20.3, NS for all groups	Cross-sectional	[79]
**IL-17a, pg/mL, median [IQR]**	NA	≈150 [50–300]	≈450 [200–1000] (**)	Cross-sectional	[80]
IL-21, pg/mL, median [IQR]	NA	≈5 [2.5–15]	≈15 [7.5–30] (#)	Cross-sectional	[80]
IL-8, pg/mL, mean (95% CI)	26.17 (24.48–27.87)	27.84 (24.37–31.30)	28.42 (23.94–32.89), NS for all groups	Cross-sectional	[55]
**IL-1** **β** **, pg/mL, mean (95% CI)**	10.09 (8.48–11.70)	10.30 (8.79–11.81)	10.11 (7.79–12.44) NS for all groups	Cross-sectional	[55]
	Cross-sectional, AUC = 0.89 to indicate metabolic syndrome				[81]
**IL-10, pg/mL, mean (95% CI)**	10.01 (8.90–11.12)	10.41 (9.11–11.71)	10.10 (8.02–12.18), NS for all groups	Cross-sectional	[55]
	Cross-sectional, AUC = 1.0 to indicate metabolic syndrome				[81]
**Lysophosphatidylcholines (Lyso-PC), μmol/L**	NA	86.34 ± 3.3	70.42 ± 2.4, (##)	Longitudinal, AUC = 0.77, for detecting MASLD	[41]
	100.55 ± 5.76	83.53 ± 2.81	84.42 ± 4.17 (# for MHO, MUO, and healthy)	Cross-sectional	[42]
**C3, mg/dL, mean ± SEM**	126.66 ± 20.88	140.45 ± 21.06	148.2 ± 23.98, (*** for all groups)	Cross-sectional	[82,83]
	Cross-sectional, AUC = 0.80 for detecting MASLD, cut-off 116.50 mg/dL				[84]
**PAI-1, ng/mL, mean ± SEM**	24.58 ± 10.73	24.79 ± 9.75	30.53 ± 12.34, (*** for MHO vs. MUO and for MHO vs. healthy)	Cross-sectional	[82,83]
	17.35 ± 4.45	NA	53.85 ± 16.45 (***)	Cross-sectional, AUC = 0.975, cut-off = 25.0 ng/mL for MUO	[85]
MCP-1, pg/mL, mean ± SD	324.7 ± 221.7	359.9 ± 210.1	346.1 ± 213.9, NS for all groups	Cross-sectional	[86]
Leptin, pg/mL, mean ± SD	12 ± 12	43 ± 12, (* MHO vs. healthy)	41 ± 9, (* MUO vs. healthy)	Cross-sectional	[53]
		36.27 ± 24.02	53.07 ± 34.56 (#)	Cross-sectional	[87]
**Leptin, ng/mL (Me [IQR])**	5.80 (4.61, 7.20) AUC = 0.77 for distinguishing healthy and MHO, AUC = 0.894 for distinguishing healthy and MUO (**)	8.82 (6.40, 11.22) AUC = 0.734 for distinguishing MHO and MUO (**) (# vs. healthy)	12.60 (9.06, 15.69) (# vs. healthy and MHO)	Cross-sectional, ROC curve analysis is available	[88]
Adiponectin, μg/mL, mean ± SD	12.1 ± 7.2	3.1 ± 1.5, (* MHO vs. healthy)	2.7 ± 1.9, (* MUO vs. healthy and MHO)	Cross-sectional	[53,89]
**Adiponectin, μg/mL, mean ± SD (Me [IQR])**	11.93 (9.77, 13.73), AUC = 0.787 for distinguishing healthy and MHO, AUC = 0.877 for distinguishing healthy and MUO (**)	9.18 (7.95, 10.61), AUC = 0.656 for distinguishing MHO and MUO (**) (# vs. healthy)	8.34 (7.17, 9.39) (# vs. healthy and MHO)	Cross-sectional, ROC curve analysis is available	[88]
Vaspin, ng/mL, mean ± SD	NA	2.07 ± 3.2	2.14 ± 2.2, NS	Cross-sectional	[90]
Visfatin, ng/mL (Me [IQR])	14,961.90 [12,397.98–20,576.90]	14,809.15 [13,715.20–19,280.44]	15,319.60 [12,696.9–17,003.08, NS for all groups.	Cross-sectional	[91]
Resistin, ng/mL, mean ± SEM	5.56 ± 3.90	5.63 ± 2.71	6.09 ± 2.74, (## for MUO vs. MHO and for MHO vs. healthy)	Cross-sectional	[82]
**Resistin, ng/mL, (Me [IQR])**	8.36 (6.84, 10.01), AUC = 0.751 for distinguishing healthy and MHO, AUC = 0.826 for distinguishing healthy and MUO (**)	11.06 (8.89, 13.32), AUC = 0.644 for distinguishing MHO and MUO (**) (# vs. healthy)	13.11 (10.01, 15.97) (# vs. healthy and MHO)	Cross-sectional, ROC curve analysis is available	[88]
Adipsin, mg/L (Me [IQR])	1.37 [0.92–1.96]	0.98 [0.81–1.24]	1.03 [0.85–1.51], # for all groups.	Cross-sectional	[91]
**Fetuin-a, μg/mL, mean ± SD**	NA	327 ± 74	377 ± 59, (##)	Cross-sectional	[90]
	NA	621.32	909.06	Cross-sectional, AUC = 0.807 for differentiation between patients with MHO and MUO (**), cut-off for T2DM ≥ 821	[92]
**Chemerin, ng/mL, mean ± SD**	NA	194 ± 33	236 ± 35, (**)	Cross-sectional	[90]
	25.21 ± 12.95	NA	50.13 ± 12.50 (**)	Cross-sectional, AUC = 0.89 (95% CI: 0.85–0.93) (**), cut-off = 250.0 pg/mL for diagnosis MUO	[93]
**Myeloperoxidase of neutrophils, ng/mL, mean ± SD**	17.3 ± 5.5	27.1 ± 10.8 (**)	NA	Cross-sectional	[94]
	22 ± 12	86 (MHO+MUO)		Cross-sectional, AUC = 0.82 for identifying of insulin resistance, cut-off = 87.8	[95]
CD66b expression (MFI, arbitrary units, mean ± SD)	129.7 ± 9.2	177.3 ± 43.7 (**)	NA	Cross-sectional	[94]
Retinol-binding protein-4, μg/mL, mean ± SD	NA	42.7 ± 23	88.6 ± 32, (**)	Cross-sectional	[90,96]
**Retinol-binding protein-4, μg/mL, (Me [IQR])**	32.96 (27.48, 37.78), AUC = 0.746 for distinguishing healthy and MHO, AUC = 0.859 for distinguishing healthy and MUO (**)	39.61 (33.98, 45.79) (# vs. healthy), AUC = 0.684 for distinguishing MHO and MUO (**)	46.25 (38.78, 53.36) (# vs. healthy and MHO)	Cross-sectional, ROC-curve analysis is available	[88]
sICAM-1, ng/mL, mean ± SD	267.8 ± 73.4	273.1 ± 78.6, (NS for MHO vs. healthy)	298.7 ± 86.2, (** for MUO vs. healthy)	Cross-sectional	[12]
**sE-selectin, ng/mL, mean ± SD**	51.2 ± 23.2	56.0 ± 22.9, (NS for MHO vs. healthy)	64.0 ± 29.9, (** MUO vs. healthy)	Cross-sectional	[12]
	9.18 ± 0.38	32.19 ± 2.01 (#)	62.79 ± 6.31 (# vs. healthy and MHO)	Cross-sectional, AUC = 0.87 for diagnosis MUO	[97]
Calprotectin, ng/mL, mean ± SD	65.1 ± 23.1	115.5 ± 43.5 (**)	NA	Cross-sectional	[94]
Fibrinogen, mg/dL (Me [IQR])	248.00 [220.00–297.00]	256.00 [234.00–278.50]	303.00 [260.25–334.00], (* for all groups)	Cross-sectional	[91]

Notes to Table 2. *—*p* < 0.01; **—*p* < 0.001; ***—*p* < 0.0001; AUC—area under the ROC curve, #—*p* < 0.05; ##—*p* < 0.005, NS—non significant. «NA» data are not available. Data marked in bold are potential predicting MUO biomarkers. Green highlighting represents the most statistically significant results with the highest potential to serve as a biomarker.

**Table 3:** Circulating immune cells in healthy individuals, MHO, and MUO.

Cell Type	Healthy Individuals	MHO	MUO	Type of Study, Biomarker Validation	References
**Neutrophil/lymphocyte ratio, mean ± SD**	1.82 ± 1.02	3.67 ± 0.95 (** for MHO+MUO vs. healthy)		Cross-sectional, cut-off for the development of T2DM = 3.12, AUC = 0.701.	[127]
Neutrophil/lymphocyte ratio, median (min, max)	1.21 (0.52, 2.09)	1.33 (0.53, 2.23) (#)	NA	Cross-sectional	[128]
**Leukocytes, ×10^3^/mm^3^, mean ± SD**	**4.000–9.000**	**NA**	**>7.100—independent risk factor for T2DM**	**Longitudinal,** **AUC = 0.59 for T2DM prediction**	[126]
Leukocytes, ×10^3^/mm^3^, mean ± SD	8.107 ± 2.022	7.877 ± 1.993—non-progressors	8.555 ± 1.780, progressors, (** for MUO vs. MHO)	Longitudinal	[14]
	6.060 ± 1.940	6.040 ± 1.660	6.580 ± 2.130, (** for all gr.)	Cross-sectional	[125]
	6.750 ± 1.800	NA	7.270 ± 2.010, (**)	Cross-sectional	[57]
Neutrophils, ×10^3^/mm^3^, mean ± SD	1.5–6.8	4.000 ± 1.440	4.350 ± 1.580, (**)	Cross-sectional	[57]
Neutrophils, ×10^3^/mm^3^, mean ± SD	4.977 ± 1.760	4.900 ± 1.727—non-progressors	5.110 ± 1.655, progressors, (NS for MHO and MUO)	Longitudinal	[14]
**Neutrophils ×10^9^ cells/L**	Longitudinal, AUC = 0.57 (95% CI: 0.55–0.59) * for indicating MUO				[76]
Lymphocytes, ×10^3^/mm^3^, mean ± SD	2.441 ± 0.078	2.373 ± 0.696—non-progressors	2.576 ± 0.813—progressors, (NS for MHO and MUO)	Longitudinal	[14]
Lymphocyte count ×10^9^ cells/L	Longitudinal, AUC = 0.54 * for indicating MUO				[76]
Monocytes, ×10^3^/mm^3^, mean ± SD	0.520 ± 0.160	NA	0.560 ± 0.220, (**)	Cross-sectional	[57]
Monocytes, ×10^3^/mm^3^, mean ± SD	0.440 ± 0.250	0.478 ± 0.235	0.352 ± 0.290, (* for MHO vs. MUO)	Longitudinal	[14]
Monocyte count ×10^9^ cells/L	Longitudinal, AUC = 0.56 * for indicating MUO				[76]
CD11b+ activated monocytes, rMFI (arbitrary units, mean ± SE)	11.2 ± 2.0	42.6 ± 9.4, (# for MUO+MHO vs. healthy)		Cross-sectional	[56]
B cells, ×10^3^/mm^3^, mean ± SD	0.260 ± 0.140	NA	0.310 ± 0.170, (** for healthy vs. MUO)	Cross-sectional	[129]
Transitional CD19+CD27+CD38high B cells, % of mononuclear cells, mean ± SD	2.4 ± 0.6	0.7 ± 0.2, (# (metabolic status not specified)	NA	Cross-sectional	[130]
CD19+CD24highCD38highIL-10+ Bregs, % of mononuclear cells, mean ± SD	6.0 ± 0.9	3.9 ± 1.4, NS (metabolic status not specified)	NA	Cross-sectional	[130]
NK cells, % of lymphocytes, mean (range)	16.6 (7.6–28.6)	NA	7.6 (2.2–19.0), * (but with BMI > 40 kg/m^2^)	Cross-sectional	[131]
NK cells, ×10^3^/mm^3^, Me [IQR]	0.100 [0.80–0.150]	0.140 [0.100–0.190], (metabolic status not specified), #	NA	Cross-sectional	[132]
NK cells, % of lymphocytes, mean ± SEM	12.3 (SEM not specified) (# for healthy versus MHO+MUO group)	11.7 ± 0.9	6.5 ± 3.1, (*** for MUO and MHO)	Cross-sectional	[20]
Natural killer T cells (CD3+, CD56+; % of PBMCs), mean ± SEM	3.9 ± 0.9	3.8 ± 1.1, NS (metabolic status not specified)	NA	Cross-sectional	[133]
CD8 cells, % of lymphocytes, mean (SE)	19.9 (SE not specified), (* for healthy vs. MHO+MUO)	13.4 (SE < 1.1)	9.3 (SE < 1.4), (# for MHO and MUO)	Cross-sectional	[20]
Th1/Th2 ratio, mean ± SE	1.0 ± 0.2	2.3 ± 0.5 (# for MHO+MUO vs. healthy)		Cross-sectional	[56]
Th17% of CD3+CD4+ lymphocytes, median [Q1; Q3]	0.041 [0.023; 0.099]	0.097 [0.044; 0.289], (# metabolic status not specified)	NA	Cross-sectional	[134]
Th1% of CD4+ lymphocytes, mean ± SE	~2.4 ± 0.8	NA	~4.7 ± 1.4 (NS for MHO+MUO vs. healthy)	Cross-sectional	[56]
Th2% of CD4+ lymphocytes, mean ± SE	~4.0 ± 1.3	~2.0 ± 0.4 (NS for MHO+MUO vs. healthy)	NA	Cross-sectional	[56]
**Treg, % of CD4+ lymphocytes, Me [IQR]**	1.2 [0.67–2.01]	0.73 [0.32–1.11] (# for MHO+MUO vs. healthy)	NA	Cross-sectional, T-reg cut-off <0.73%, AUC = 0.75 for HbA1c *≥* 5.5%	[58]
**Treg/Th17 ratio, Me [IQR]**	2.59 (1.71, 5.29)	2.24 (1.32, 3.02)	1.18 (0.78, 2.31) (# vs. healthy and vs. MHO)	Cross-sectional, AUC = 0.73 when predicting glucose metabolic status	[135]
CD4+FOXP3highCD45RA- % of CD4+ lymphocytes, mean ± SEM	~1.3 ± 0.14	~0.9 ± 0.07, (# metabolic status not specified)	NA	Cross-sectional	[136]
Eosinophils, ×10^3^/mm^3^, mean ± SD	0.196 ± 0.157	0.189 ± 0.155—non-progressors	0.225 ± 0.204—progressors, (NS for MHO and MUO)	Longitudinal	[14]
Basophils, ×10^3^/mm^3^, mean ± SD	0.040 ± 0.052	0.047 ± 0.006—non-progressors	0.047 ± 0.013—progressors, (NS for MHO and MUO)	Longitudinal	[14]

Notes for Table 3. *—*p* < 0.01; **—*p* < 0.001; ***—*p* < 0.0001; AUC—area under the ROC curve, #—*p* < 0.05; NS—non significant, rMFI—relative mean fluorescence intensity. «NA» Data are not available. Data marked in bold are potential predicting MUO biomarkers. Green highlight represents the most statistically significant results with the highest potential to serve as a biomarker.

## Data Availability

No new data were created or analyzed in this study.

## Supplementary Materials

The following supporting information can be downloaded at https://www.mdpi.com/article/10.3390/biomedicines14051161/s1, Table S1: Anthropometric and biochemical characteristics of healthy individuals and in MHO and MUO.

## Abbreviations

AGEs	Advanced glycation end products
AT	Adipose tissue
ATI	Adipose tissue inflammation
AMPK	AMP-activated protein kinase
ATP	Adenosine triphosphate
ABCA1	ATP-binding cassette subfamily A member 1
ACC1	Acetyl-CoA carboxylase 1
BMI	Body mass index
BPD	Biliopancreatic diversion
BS	Bariatric surgery
CXCL	C–X–C motif chemokine ligand
CRP	C-reactive protein
CCL	Chemokine (CC motif) ligand
CCR6	C-C chemokine receptor type 6
Csf1r	Colony stimulating factor 1 receptor
DAMPs	Danger-associated molecular patterns
DNA	Deoxyribonucleic acid
EWL	Excess weight loss
ECM	Extracellular matrix
ER	Endoplasmic reticulum
FABP7	Fatty acid binding protein 7
FFAs	Free fatty acids
FAS	Fatty acid synthesis
FAO	Fatty acid oxidation
GC	Germinal center
HMGB1	High-mobility group protein B1
HIF1a	Hypoxia inducible factor 1α
ILC	Innate lymphoid cell
IR	Insulin resistance
IRS	Insulin receptor substrate
IL	Interleukin
iCAM	Intercellular adhesion molecule
IFN	Interferon
IGFBP3	Insulin-like growth factor binding protein 3
JNK	C-Jun N-terminal kinase
LTB4R	Leukotriene B4 receptor
MSCs	Mesenchymal stem cells
METRNL	Meteorin-like hormone
MHO	Metabolically “healthy” obesity
MUO	Metabolically “unhealthy” obesity
MIP	Macrophage inflammatory protein
MAFLD	Metabolic-associated fatty liver disease
MCP-1	Monocyte chemoattractant protein 1
MHC	Major histocompatibility complex
mTOR	Mechanistic target of rapamycin kinase
NLPR3	NLR family pyrin domain containing 3
NF-kB	Nuclear factor kappa-light-chain-enhancer of activated B cells
NLR	Neutrophil to lymphocyte ratio
NO	Nitric oxide
NETs	Neutrophil extracellular traps
OXPHOS	Oxidative phosphorylation
OAGB	One anastomosis gastric bypass
PAI-1	Plasminogen activator inhibitor-1
RAGE	Receptor for advanced glycation end products
ROS	Reactive oxygen species
RYGB	Ry-gastric bypass
SAT	Subcutaneous adipose tissue
SASP	Senescence-associated secretory phenotype
SG	Sleeve gastrectomy
SADI	Single anastomosis duodeno-Ileal bypass
T2DM	Type 2 diabetes mellitus
TNF-a	Tumor necrosis factor-a
TGF	Transforming growth factor
TLR	Toll-like receptor
TCR	T-cell receptor
TFAM	Mitochondrial transcription factor
UPR	Unfolded protein response
UCP-1	Uncoupling protein 1
VAT	Visceral adipose tissue
VEGF	Vascular endothelial growth factor
vCAM	Vascular cell adhesion molecule

## References

[1] Mayoral L.P., Andrade G.M., Mayoral E.P., Huerta T.H., Canseco S.P., Rodal Canales F.J., Cabrera-Fuentes H.A., Cruz M.M., Santiago A.D.P., Alpuche J.J. (2020). Obesity subtypes, related biomarkers & heterogeneity. Indian J. Med. Res..

[2] Rubino F., Cummings D.E., Eckel R.H., Cohen R.V., Wilding J.P.H., Brown W.A., Stanford F.C., Batterham R.L., Farooqi I.S., Farpour-Lambert N.J. (2025). Definition and diagnostic criteria of clinical obesity. Lancet Diabetes Endocrinol..

[3] Soriguer F., Gutiérrez-Repiso C., Rubio-Martín E., García-Fuentes E., Almaraz M.C., Colomo N., de Antonio I.E., de Adana M.S.R., Chaves F.J., Morcillo S. (2013). Metabolically healthy but obese, a matter of time? Findings from the prospective Pizarra study. J. Clin. Endocrinol. Metab..

[4] Appleton S.L., Seaborn C.J., Visvanathan R., Hill C.L., Gill T.K., Taylor A.W., Adams R.J., On Behalf of The North West Adelaide Health Study Team (2013). Diabetes and cardiovascular disease outcomes in the metabolically healthy obese phenotype: A cohort study. Diabetes Care.

[5] Ler P., Ojalehto E., Zhan Y., Finkel D., Dahl Aslan A.K., Karlsson I.K. (2024). Conversions between metabolically unhealthy and healthy obesity from midlife to late-life. Int. J. Obes..

[6] Hwang Y.C., Hayashi T., Fujimoto W.Y., Kahn S.E., Leonetti D.L., McNeely M.J., Boyko E.J. (2015). Visceral abdominal fat accumulation predicts the conversion of metabolically healthy obese subjects to an unhealthy phenotype. Int. J. Obes..

[7] Smith G.I., Mittendorfer B., Klein S. (2019). Metabolically healthy obesity: Facts and fantasies. J. Clin. Investig..

[8] Stefan N., Kantartzis K., Machann J., Schick F., Thamer C., Rittig K., Balletshofer B., Machicao F., Fritsche A., Häring H.-U. (2008). Identification and characterization of metabolically benign obesity in humans. Arch. Intern. Med..

[9] Iacobini C., Pugliese G., Blasetti Fantauzzi C., Federici M., Menini S. (2019). Metabolically healthy versus metabolically unhealthy obesity. Metabolism.

[10] Ruggiero A.D., Key C.C., Kavanagh K. (2021). Adipose Tissue Macrophage Polarization in Healthy and Unhealthy Obesity. Front. Nutr..

[11] Zhao J.Y., Zhou L.J., Ma K.L., Hao R., Li M. (2024). MHO or MUO? White Adipose Tissue Remodeling. Obes. Rev..

[12] Liu M., Wang P., Xie P., Xu X., He L., Chen X., Zhang S., Lin Y., Huang Y., Xia W. (2023). Expression of ICAM-1 and E-selectin in different metabolic obesity phenotypes: Discrepancy for endothelial dysfunction. J. Endocrinol. Investig..

[13] Su Z., Efremov L., Mikolajczyk R. (2024). Differences in the levels of inflammatory markers between metabolically healthy obese and other obesity phenotypes in adults: A systematic review and meta-analysis. Nutr. Metab. Cardiovasc. Dis..

[14] Vozarova B., Weyer C., Lindsay R.S., Pratley R.E., Bogardus C., Tataranni P.A. (2002). High white blood cell count is associated with a worsening of insulin sensitivity and predicts the development of type 2 diabetes. Diabetes.

[15] Blüher M. (2020). Metabolically Healthy Obesity. Endocr. Rev..

[16] Cătoi A.F., Busetto L. (2019). Metabolically Healthy Obesity and Bariatric Surgery. Obes. Surg..

[17] Tanriover C., Copur S., Gaipov A., Ozlusen B., Akcan R.E., Kuwabara M., Hornum M., Van Raalte D.H., Kanbay M. (2023). Metabolically healthy obesity: Misleading phrase or healthy phenotype?. Eur. J. Intern. Med..

[18] Duque A.P., Rodrigues Junior L.F., Mediano M.F.F., Tibiriça E., De Lorenzo A. (2020). Emerging concepts in metabolically healthy obesity. Am. J. Cardiovasc. Dis..

[19] Lin H., Zhang L., Zheng R., Zheng Y. (2017). The prevalence, metabolic risk and effects of lifestyle intervention for metabolically healthy obesity: A systematic review and meta-analysis: A PRISMA-compliant article. Medicine.

[20] Lynch L.A., O’Connell J.M., Kwasnik A.K., Cawood T.J., O’Farrelly C., O’Shea D.B. (2009). Are natural killer cells protecting the metabolically healthy obese patient?. Obesity.

[21] Primeau V., Coderre L., Karelis A.D., Brochu M., Lavoie M.-E., Messier V., Sladek R., Rabasa-Lhoret R. (2011). Characterizing the profile of obese patients who are metabolically healthy. Int. J. Obes..

[22] Lee K. (2009). Metabolically obese but normal weight (MONW) and metabolically healthy but obese (MHO) phenotypes in Koreans: Characteristics and health behaviors. Asia Pac. J. Clin. Nutr..

[23] Moussa O., Arhi C., Ziprin P., Darzi A., Khan O., Purkayastha S. (2019). Fate of the metabolically healthy obese-is this term a misnomer? A study from the Clinical Practice Research Datalink. Int. J. Obes..

[24] Eckel N., Li Y., Kuxhaus O., Stefan N., Hu F.B., Schulze M.B. (2018). Transition from metabolic healthy to unhealthy phenotypes and association with cardiovascular disease risk across BMI categories in 90 257 women (the Nurses’ Health Study): 30 year follow-up from a prospective cohort study. Lancet Diabetes Endocrinol..

[25] Kabat G.C., Wu W.Y.-Y., Bea J.W., Chen C., Qi L., Stefanick M.L., Chlebowski R.T., Lane D.S., Wactawski-Wende J., Wassertheil-Smoller S. (2017). Metabolic phenotypes of obesity: Frequency, correlates and change over time in a cohort of postmenopausal women. Int. J. Obes..

[26] Lee D.C., Sui X., Artero E.G., Lee I.M., Church T.S., McAuley P.A., Stanford F.C., Kohl H.W., Blair S.N. (2011). Long-term effects of changes in cardiorespiratory fitness and body mass index on all-cause and cardiovascular disease mortality in men: The Aerobics Center Longitudinal Study. Circulation.

[27] Stefan N., Schick F., Häring H.U. (2017). Causes, Characteristics, and Consequences of Metabolically Unhealthy Normal Weight in Humans. Cell Metab..

[28] McLaughlin T., Lamendola C., Liu A., Abbasi F. (2011). Preferential fat deposition in subcutaneous versus visceral depots is associated with insulin sensitivity. J. Clin. Endocrinol. Metab..

[29] Stafeev I., Podkuychenko N., Michurina S., Sklyanik I., Panevina A., Shestakova E., Yah’yaev K., Fedenko V., Ratner E., Vorotnikov A. (2019). Low proliferative potential of adipose-derived stromal cells associates with hypertrophy and in fl ammation in subcutaneous and omental adipose tissue of patients with type 2 diabetes mellitus. J. Diabetes Complicat..

[30] Patel P., Abate N. (2013). Body fat distribution and insulin resistance. Nutrients.

[31] Deng X., Qiu L., Sun X., Li H., Chen Z., Huang M., Hu F., Zhang Z. (2023). Early prediction of body composition parameters on metabolically unhealthy in the Chinese population via advanced machine learning. Front. Endocrinol..

[32] Hotamisligil G.S. (2006). Inflammation and metabolic disorders. Nature.

[33] Recarte M., Corripio R., Palma S., Mata A., de-Cos A.I. (2023). Improvement of Low-Grade Inflammation in Patients with Metabolically Healthy Severe Obesity After Primary Bariatric Surgery. Obes. Surg..

[34] Castro A.M., Macedo-de la Concha L.E., Pantoja-Meléndez C.A. (2017). Low-grade inflammation and its relation to obesity and chronic degenerative diseases. Rev. Médica Hosp. Gen. México.

[35] Alam I., Ng T.P., Larbi A. (2012). Does inflammation determine whether obesity is metabolically healthy or unhealthy? The aging perspective. Mediat. Inflamm..

[36] Wu H., Ballantyne C.M. (2020). Metabolic Inflammation and Insulin Resistance in Obesity. Circ. Res..

[37] Shin M.J., Hyun Y.J., Kim O.Y., Kim J.Y., Jang Y., Lee J.H. (2006). Weight loss effect on inflammation and LDL oxidation in metabolically healthy but obese (MHO) individuals: Low inflammation and LDL oxidation in MHO women. Int. J. Obes..

[38] Marques-Vidal P., Velho S., Waterworth D., Waeber G., von Känel R., Vollenweider P. (2012). The association between inflammatory biomarkers and metabolically healthy obesity depends of the definition used. Eur. J. Clin. Nutr..

[39] You T., Nicklas B.J., Ding J., Penninx B.W.J.H., Goodpaster B.H., Bauer D.C., Tylavsky F.A., Harris T.B., Kritchevsky S.B. (2008). The metabolic syndrome is associated with circulating adipokines in older adults across a wide range of adiposity. J. Gerontol. A Biol. Sci. Med. Sci..

[40] Karelis A.D., Faraj M., Bastard J.-P., St-Pierre D.H., Brochu M., Prud’homme D., Rabasa-Lhoret R. (2005). The metabolically healthy but obese individual presents a favorable inflammation profile. J. Clin. Endocrinol. Metab..

[41] Lehmann R., Franken H., Dammeier S., Rosenbaum L., Kantartzis K., Peter A., Zell A., Adam P., Li J., Xu G. (2013). Circulating lysophosphatidylcholines are markers of a metabolically benign nonalcoholic fatty liver. Diabetes Care.

[42] Barber M.N., Risis S., Yang C., Meikle P.J., Staples M., Febbraio M.A., Bruce C.R. (2012). Plasma lysophosphatidylcholine levels are reduced in obesity and type 2 diabetes. PLoS ONE.

[43] Stienstra R., Stefan N. (2013). Tipping the inflammatory balance: Inflammasome activation distinguishes metabolically unhealthy from healthy obesity. Diabetologia.

[44] Mai Z., Chen Y., Mao H., Wang L. (2024). Association between the skeletal muscle mass to visceral fat area ratio and metabolic dysfunction-associated fatty liver disease: A cross-sectional study of NHANES 2017–2018. J. Diabetes.

[45] Lonardo A., Mantovani A., Lugari S., Targher G. (2020). Epidemiology and pathophysiology of the association between NAFLD and metabolically healthy or metabolically unhealthy obesity. Ann. Hepatol..

[46] Ampuero J., Aller R., Gallego-Durán R., Banales J.M., Crespo J., García-Monzón C., Pareja M.J., Vilar-Gómez E., Caballería J., Escudero-García D. (2018). The effects of metabolic status on non-alcoholic fatty liver disease-related outcomes, beyond the presence of obesity. Aliment Pharmacol. Ther..

[47] Kim H.K., Lee M.J., Kim E.H., Bae S.J., Kim K.W., Kim C.H. (2021). Comparison of muscle mass and quality between metabolically healthy and unhealthy phenotypes. Obesity.

[48] Molli A.E.I., Steinhardt A.P., López A.P., González C.D., Vilariño J., Frechtel G.D., Cerrone G.E. (2017). Metabolically healthy obese individuals present similar chronic inflammation level but less insulin-resistance than obese individuals with metabolic syndrome. PLoS ONE.

[49] Efremov L., Lacruz M.E., Tiller D., Medenwald D., Greiser K.H., Kluttig A., Wienke A., Nuding S., Mikolajczyk R. (2020). Metabolically Healthy, but Obese Individuals and Associations with Echocardiographic Parameters and Inflammatory Biomarkers: Results from the CARLA Study. Diabetes Metab. Syndr. Obes..

[50] Liao C., Gao W., Cao W., Lv J., Yu C., Wang S., Pang Z., Cong L., Wang H., Wu X. (2021). Associations of Metabolic/Obesity Phenotypes with Insulin Resistance and C-Reactive Protein: Results from the CNTR Study. Diabetes Metab. Syndr. Obes..

[51] Kalra S., Raizada N. (2024). Dyslipidemia in diabetes. Indian Heart J..

[52] Poznyak A., Grechko A.V., Poggio P., Myasoedova V.A., Alfieri V., Orekhov A.N. (2020). The Diabetes Mellitus-Atherosclerosis Connection: The Role of Lipid and Glucose Metabolism and Chronic Inflammation. Int. J. Mol. Sci..

[53] Mulhem A., Moulla Y., Klöting N., Ebert T., Tönjes A., Fasshauer M., Dietrich A., Schön M.R., Stumvoll M., Richter V. (2021). Circulating cell adhesion molecules in metabolically healthy obesity. Int. J. Obes..

[54] Brant L.C.C., Wang N., Ojeda F.M., LaValley M., Barreto S.M., Benjamin E.J., Mitchell G.F., Vasan R.S., Palmisano J.N., Münzel T. (2017). Relations of Metabolically Healthy and Unhealthy Obesity to Digital Vascular Function in Three Community-Based Cohorts: A Meta-Analysis. J. Am. Heart Assoc..

[55] Ferreira F.G., Reitz L.K., Valmorbida A., Papini Gabiatti M., Hansen F., Faria Di Pietro P., Licursi de Oliveira L., Santos de Moraes Trindade E.B., Zarbato Longo G. (2022). Metabolically unhealthy and overweight phenotypes are associated with increased levels of inflammatory cytokines: A population-based study. Nutrition.

[56] Viardot A., Heilbronn L.K., Samocha-Bonet D., Mackay F., Campbell L.V., Samaras K. (2012). Obesity is associated with activated and insulin resistant immune cells. Diabetes Metab. Res. Rev..

[57] Li Z., Yao Z., Liu Q. (2025). The association between white blood cell counts and metabolic health obesity among US adults. Front. Nutr..

[58] Wagner N., Brandhorst G., Czepluch F., Lankeit M., Eberle C., Herzberg S., Faustin V., Riggert J., Oellerich M., Hasenfuss G. (2013). Circulating regulatory T cells are reduced in obesity and may identify subjects at increased metabolic and cardiovascular risk. Obesity.

[59] An S.M., Cho S.H., Yoon J.C. (2023). Adipose Tissue and Metabolic Health. Diabetes Metab. J..

[60] Chait A., den Hartigh L.J. (2020). Adipose Tissue Distribution, Inflammation and Its Metabolic Consequences, Including Diabetes and Cardiovascular Disease. Front. Cardiovasc. Med..

[61] Cobos-Palacios L., Ruiz-Moreno M.I., Vilches-Perez A., Vargas-Candela A., Muñoz-Úbeda M., Porres J.B., Navarro-Sanz A., Lopez-Carmona M.D., Sanz-Canovas J., Perez-Belmonte L.M. (2022). Metabolically healthy obesity: Inflammatory biomarkers and adipokines in elderly population. PLoS ONE.

[62] Barbarroja N., López-Pedrera R., Mayas M.D., García-Fuentes E., Garrido-Sánchez L., Macías-González M., El Bekay R., Vidal-Puig A., Tinahones F.J. (2010). The obese healthy paradox: Is inflammation the answer?. Biochem. J..

[63] Lawrence T., Gilroy D.W. (2007). Chronic inflammation: A failure of resolution?. Int. J. Exp. Pathol..

[64] Nogueira T. Chronic Low-Grade Inflammation. Encyclopedia. https://encyclopedia.pub/entry/16988.

[65] Makki K., Froguel P., Wolowczuk I. (2013). Adipose tissue in obesity-related inflammation and insulin resistance: Cells, cytokines, and chemokines. ISRN Inflamm..

[66] Blüher M. (2016). Adipose tissue inflammation: A cause or consequence of obesity-related insulin resistance?. Clin. Sci..

[67] Andersen C.J., Murphy K.E., Fernandez M.L. (2016). Impact of Obesity and Metabolic Syndrome on Immunity. Adv. Nutr..

[68] Deng J., Liu S., Zou L., Xu C., Geng B., Xu G. (2012). Lipolysis response to endoplasmic reticulum stress in adipose cells. J. Biol. Chem..

[69] Pirola L., Ferraz J.C. (2017). Role of pro- and anti-inflammatory phenomena in the physiopathology of type 2 diabetes and obesity. World J. Biol. Chem..

[70] Khanna D., Khanna S., Khanna P., Kahar P., Patel B.M. (2022). Obesity: A Chronic Low-Grade Inflammation and Its Markers. Cureus.

[71] Uribe-Querol E., Rosales C. (2022). Neutrophils Actively Contribute to Obesity-Associated Inflammation and Pathological Complications. Cells.

[72] Ouchi N., Parker J.L., Lugus J.J., Walsh K. (2011). Adipokines in inflammation and metabolic disease. Nat. Rev. Immunol..

[73] Zhao L., Ni Y., Ma X., Zhao A., Bao Y., Liu J., Chen T., Xie G., Panee J., Su M. (2016). A panel of free fatty acid ratios to predict the development of metabolic abnormalities in healthy obese individuals. Sci. Rep..

[74] Sánchez E., Baena-Fustegueras J.A., de la Fuente M.C., Gutiérrez L., Bueno M., Ros S., Lecube A. (2017). Productos finales deglicación avanzada en la obesidad mórbida y tras la cirugía bariátrica: Cuando la memoria glucémica empieza a fallar. Endocrinol. Diabetes Nutr..

[75] Zahedi A.S., Daneshpour M.S., Akbarzadeh M., Hedayati M., Azizi F., Zarkesh M. (2023). Association of baseline and changes in adiponectin, homocysteine, high-sensitivity C-reactive protein, interleukin-6, and interleukin-10 levels and metabolic syndrome incidence: Tehran lipid and glucose study. Heliyon.

[76] Zhao L., Cui M., Yang S., Zhou H., Li M. (2024). Systemic Inflammatory Indicators and Risk of Incident Metabolically Unhealthy Phenotype. J. Inflamm. Res..

[77] Mohany K., Al Rugaie O., Al-Wutayd O., Alsharidah M., Al-Nafeesah A. (2022). Circulating miR-15b, Annexin A1, procalcitonin and interleukin-6 levels differentiate children with metabolically unhealthy obesity from those with metabolically healthy obesity: A case-control study. Exp. Ther. Med..

[78] Tylutka A., Morawin B., Walas Ł., Michałek M., Gwara A., Zembron-Lacny A. (2023). Assessment of metabolic syndrome predictors in relation to inflammation and visceral fat tissue in older adults. Sci. Rep..

[79] Perreault M., Zulyniak M.A., Badoud F., Stephenson S., Badawi A., Buchholz A., Mutch D.M. (2014). A distinct fatty acid profile underlies the reduced inflammatory state of metabolically healthy obese individuals. PLoS ONE.

[80] Ip B., Cilfone N.A., Belkina A.C., DeFuria J., Jagannathan-Bogdan M., Zhu M., Kuchibhatla R., McDonnell M.E., Xiao Q., Kepler T.B. (2016). Th17 cytokines differentiate obesity from obesity-associated type 2 diabetes and promote TNFα production. Obesity.

[81] Sanches M.D., Goldberg T.B.L., Rizzo A.d.C.B., da Silva V.N., Mosca L.N., Romagnoli G.G., Gorgulho C.M., Junior J.P.A., de Lima G.R., Betti I.R. (2023). Inflammatory cytokines and chemokines in obese adolescents with antibody against to adenovirus 36. Sci. Rep..

[82] Phillips C.M., Perry I.J. (2013). Does inflammation determine metabolic health status in obese and nonobese adults?. J. Clin. Endocrinol. Metab..

[83] Wildman R.P., Kaplan R., Manson J.E., Rajkovic A., Connelly S.A., Mackey R.H., Tinker L.F., Curb J.D., Eaton C.B., Wassertheil-Smoller S. (2011). Body size phenotypes and inflammation in the Women’s Health Initiative Observational Study. Obesity.

[84] Ye D., Ma H., Zhou J., Wang J., Shi J., Chen J., Bao Z., Hu X. (2025). Serum complement C3 as a diagnostic biomarker for metabolic dysfunction -associated steatotic liver disease in middle-aged and elderly adults: A cross-sectional study. BMC Gastroenterol..

[85] Garg M., Dutta M., Mahalle N. (2012). Adipokines (adiponectin and plasminogen activator inhhibitor-1) in metabolic syndrome. Indian J. Endocrinol. Metab..

[86] Lee S.H. (2015). Adipokine Profiles and Metabolic Health. Endocrinol. Metab..

[87] Jamar G., Caranti D.A., de Cassia Cesar H., Masquio D.C.L., Bandoni D.H., Pisani L.P. (2017). Leptin as a cardiovascular risk marker in metabolically healthy obese: Hyperleptinemia in metabolically healthy obese. Appetite.

[88] Li J., Shi H., Xie Q., Zhang P. (2026). Adipose-inflammatory factor profiles in children with metabolically healthy obesity and their correlation with NAFLD severity. Front. Pediatr..

[89] Garrido O.S., Adames X.H., Torres-Atencio I., De Ycaza A.E., Arce M.F.P., Espinosa A.T., Arteaga G. (2025). Evaluation of Adiponectin as a Metabolic Risk Indicator in the Panamanian Population. Obesities.

[90] Klöting N., Fasshauer M., Dietrich A., Kovacs P., Schön M.R., Kern M., Stumvoll M., Blüher M. (2010). Insulin-sensitive obesity. Am. J. Physiol. Endocrinol. Metab..

[91] Lejawa M., Osadnik K., Czuba Z., Osadnik T., Pawlas N. (2021). Association of Metabolically Healthy and Unhealthy Obesity Phenotype with Markers Related to Obesity, Diabetes among Young, Healthy Adult Men. Analysis of MAGNETIC Study. Life.

[92] Karamfilova V., Nedeva I., Gatev T., Gateva A., Assyov Y., Gerganova A., Popov D., Velikova T., Kamenov Z. (2023). Relationship of serum Fetuin-A with metabolic and vascular parameters in patients with prediabetes and type 2 diabetes mellitus. Pharmacia.

[93] Naik B., Acharya M., Tirkey N.B., Bhoi S., Murmu M. (2025). Serum chemerin as a biomarker of metabolic syndrome. Eur. J. Cardiovasc. Med..

[94] Nijhuis J., Rensen S.S., Slaats Y., van Dielen F.M., Buurman W.A., Greve J.W. (2009). Neutrophil activation in morbid obesity, chronic activation of acute inflammation. Obesity.

[95] Zaki M., Basha W., Reyad H., Mohamed R., Hassan N., Kholousi S. (2018). Association between myeloperoxidase levels and risk of insulin resistance in Egyptian obese women. Open Access Maced. J. Med. Sci..

[96] Fu J., Li Y., Esangbedo I.C., Li G., Feng D., Li L., Xu L., Han L., Li M., Li C. (2018). Circulating Osteonectin and Adipokine Profiles in Relation to Metabolically Healthy Obesity in Chinese Children: Findings From BCAMS. J. Am. Heart Assoc..

[97] Abulnaja K.O., Kannan K., Al-Manzlawi A.M.K., Kumosani T.A., Qari M., Moselhy S.S. (2022). Sensitivity, specificity of biochemical markers for early prediction of endotheliadysfunction in atherosclerotic obese subjects. Afr. Health Sci..

[98] Ceperuelo-Mallafré V., Llauradó G., Keiran N., Benaiges E., Astiarraga B., Martínez L., Pellitero S., González-Clemente J.M., Rodríguez A., Fernández-Real J.M. (2019). Preoperative Circulating Succinate Levels as a Biomarker for Diabetes Remission After Bariatric Surgery. Diabetes Care.

[99] Alvarenga L., Cardozo L.F.M.F., Ribeiro M., Kussi F., Esgalhado M., Mafra D. (2025). Bioactive Compounds as Modulators of N-Formyl Peptide Signaling in Chronic Diseases. Molecules.

[100] Alrouji M., Al-Kuraishy H.M., Al-Gareeb A.I., Alexiou A., Papadakis M., Saad H.M., Batiha G.E.-S. (2023). The potential role of human islet amyloid polypeptide in type 2 diabetes mellitus and Alzheimer’s diseases. Diabetol. Metab. Syndr..

[101] Zhang X., Mosser D.M. (2008). Macrophage activation by endogenous danger signals. J. Pathol..

[102] Bremer A.A., Jialal I. (2013). Adipose tissue dysfunction in nascent metabolic syndrome. J. Obes..

[103] Altintas M.M., Azad A., Nayer B., Contreras G., Zaias J., Faul C., Reiser J., Nayer A. (2011). Mast cells, macrophages, and crown-like structures distinguish subcutaneous from visceral fat in mice. J. Lipid Res..

[104] Wang Y., Zhong J., Zhang X., Liu Z., Yang Y., Gong Q., Ren B. (2016). The Role of HMGB1 in the Pathogenesis of Type 2 Diabetes. J. Diabetes Res..

[105] Kim H.S. (2013). Role of insulin-like growth factor binding protein-3 in glucose and lipid metabolism. Ann. Pediatr. Endocrinol. Metab..

[106] Guerreiro V.A., Carvalho D., Freitas P. (2022). Obesity, Adipose Tissue, and Inflammation Answered in Questions. J. Obes..

[107] Claycombe K., King L.E., Fraker P.J. (2008). A role for leptin in sustaining lymphopoiesis and myelopoiesis. Proc. Natl. Acad. Sci. USA.

[108] Doumatey A.P., Zhou J., Zhou M., Prieto D., Rotimi C.N., Adeyemo A. (2016). Proinflammatory and lipid biomarkers mediate metabolically healthy obesity: A proteomics study. Obesity.

[109] Olveira A., Augustin S., Benlloch S., Ampuero J., Suárez-Pérez J.A., Armesto S., Vilarrasa E., Belinchón-Romero I., Herranz P., Crespo J. (2023). The Essential Role of IL-17 as the Pathogenetic Link between Psoriasis and Metabolic-Associated Fatty Liver Disease. Life.

[110] Lee Y.S., Olefsky J. (2021). Chronic tissue inflammation and metabolic disease. Genes Dev..

[111] Winer D.A., Winer S., Dranse H.J., Lam T.K. (2017). Immunologic impact of the intestine in metabolic disease. J. Clin. Investig..

[112] Guillemot-Legris O., Muccioli G.G. (2017). Obesity-Induced Neuroinflammation: Beyond the Hypothalamus. Trends Neurosci..

[113] Gustafson B., Nerstedt A., Spinelli R., Beguinot F., Smith U. (2022). Type 2 Diabetes, Independent of Obesity and Age, Is Characterized by Senescent and Dysfunctional Mature Human Adipose Cells. Diabetes.

[114] Mongraw-Chaffin M., Foster M.C., Anderson C.A.M., Burke G.L., Haq N., Kalyani R.R., Ouyang P., Sibley C.T., Tracy R., Woodward M. (2018). Metabolically Healthy Obesity, Transition to Metabolic Syndrome, and Cardiovascular Risk. J. Am. Coll. Cardiol..

[115] Mohamadi A., Shiraseb F., Mirzababaei A., Barekzai A.M., Clark C.C.T., Aali Y., Mirzaei K. (2023). Inflammatory markers may mediate the relationship between processed meat consumption and metabolic unhealthy obesity in women: A cross sectional study. Sci. Rep..

[116] Javeed N., Matveyenko A.V. (2018). Circadian Etiology of Type 2 Diabetes Mellitus. Physiology.

[117] Sonnweber T., Pizzini A., Nairz M., Weiss G., Tancevski I. (2018). Arachidonic Acid Metabolites in Cardiovascular and Metabolic Diseases. Int. J. Mol. Sci..

[118] ISSobczak A., ABlindauer C., JStewart A. (2019). Changes in Plasma Free Fatty Acids Associated with Type-2 Diabetes. Nutrients.

[119] Eriksen F., Carlsson E.R., Munk J.K., Madsbad S., Fenger M. (2021). Fractionated free fatty acids and their relation to diabetes status after Roux-en-Y gastric bypass: A cohort study. Physiol. Rep..

[120] Shen C.-Y., Lu C.-H., Cheng C.-F., Li K.-J., Kuo Y.-M., Wu C.-H., Liu C.-H., Hsieh S.-C., Tsai C.-Y., Yu C.-L. (2024). Advanced Glycation End-Products Acting as Immunomodulators for Chronic Inflammation, Inflammaging and Carcinogenesis in Patients with Diabetes and Immune-Related Diseases. Biomedicines.

[121] Siddiqui K., George T.P., Mujammami M., Isnani A., Alfadda A.A. (2023). The association of cell adhesion molecules and selectins (VCAM-1, ICAM-1, E-selectin, L-selectin, and P-selectin) with microvascular complications in patients with type 2 diabetes: A follow-up study. Front. Endocrinol..

[122] Pecht T., Gutman-Tirosh A., Bashan N., Rudich A. (2014). Peripheral blood leucocyte subclasses as potential biomarkers of adipose tissue inflammation and obesity subphenotypes in humans. Obes. Rev..

[123] Gu Y., Hu K., Huang Y., Zhang Q., Liu L., Meng G., Wu H., Xia Y., Bao X., Shi H. (2018). White blood cells count as an indicator to identify whether obesity leads to increased risk of type 2 diabetes. Diabetes Res. Clin. Pract..

[124] Herishanu Y., Rogowski O., Polliack A., Marilus R. (2006). Leukocytosis in obese individuals: Possible link in patients with unexplained persistent neutrophilia. Eur. J. Haematol..

[125] Yuan Y., Sun W., Kong X. (2022). Relationship between metabolically healthy obesity and the development of hypertension: A nationwide population-based study. Diabetol. Metab. Syndr..

[126] Twig G., Afek A., Shamiss A., Derazne E., Tzur D., Gordon B., Tirosh A. (2013). White blood cells count and incidence of type 2 diabetes in young men. Diabetes Care.

[127] Yilmaz H., Ucan B., Sayki M., Unsal I., Sahin M., Ozbek M., Delibasi T. (2015). Usefulness of the neutrophil-to-lymphocyte ratio to prediction of type 2 diabetes mellitus in morbid obesity. Diabetes Metab. Syndr..

[128] Aydin M., Yilmaz A., Donma M.M., Tulubas F., Demirkol M., Erdogan M., Gurel A. (2015). Neutrophil/lymphocyte ratio in obese adolescents. North. Clin. Istanb..

[129] Phillips A.C., Carroll D., Gale C.R., Drayson M., Thomas G.N., Batty G.D. (2010). Lymphocyte sub-population cell counts are associated with the metabolic syndrome and its components in the Vietnam Experience Study. Atherosclerosis.

[130] García-Hernández M., Rodríguez-Varela E., García-Jacobo R., la Torre M.H.-D., Uresti-Rivera E., González-Amaro R., Portales-Pérez D. (2018). Frequency of regulatory B cells in adipose tissue and peripheral blood from individuals with overweight, obesity and normal-weig. Obes. Res. Clin. Pract..

[131] O’Shea D., Cawood T.J., O’Farrelly C., Lynch L. (2010). Natural killer cells in obesity: Impaired function and increased susceptibility to the effects of cigarette smoke. PLoS ONE.

[132] Wouters K., Kusters Y.H., Bijnen M., Wetzels S., Zhang X., Linssen P.B., Gaens K., Houben A.J., Joris P.J., Plat J. (2020). NK cells in human visceral adipose tissue contribute to obesity-associated insulin resistance through low-grade inflammation. Clin. Transl. Med..

[133] Bähr I., Jahn J., Zipprich A., Pahlow I., Spielmann J., Kielstein H. (2018). Impaired natural killer cell subset phenotypes in human obesity. Immunol. Res..

[134] Artemniak-Wojtowicz D., Kucharska A.M., Stelmaszczyk-Emmel A., Majcher A., Pyrżak B. (2022). Changes of Peripheral Th17 Cells Subset in Overweight and Obese Children After Body Weight Reduction. Front. Endocrinol..

[135] Wen J., Liu Q., Liu M., Wang B., Li M., Wang M., Shi X., Liu H., Wu J. (2021). Increasing Imbalance of Treg/Th17 Indicates More Severe Glucose Metabolism Dysfunction in Overweight/obese Patients. Arch. Med. Res..

[136] Donninelli G., Del Cornò M., Pierdominici M., Scazzocchio B., Varì R., Varano B., Pacella I., Piconese S., Barnaba V., D’aRchivio M. (2017). Distinct Blood and Visceral Adipose Tissue Regulatory T Cell and Innate Lymphocyte Profiles Characterize Obesity and Colorectal Cancer. Front. Immunol..

[137] Friedrich K., Sommer M., Strobel S., Thrum S., Blüher M., Wagner U., Rossol M. (2019). Perturbation of the Monocyte Compartment in Human Obesity. Front. Immunol..

[138] Babio N., Ibarrola-Jurado N., Bulló M., Martínez-González M.Á., Wärnberg J., Salaverría I., Ortega-Calvo M., Estruch R., Serra-Majem L., Covas M.I. (2013). White blood cell counts as risk markers of developing metabolic syndrome and its components in the PREDIMED study. PLoS ONE.

[139] Weisberg S.P., McCann D., Desai M., Rosenbaum M., Leibel R.L., Ferrante A.W. (2003). Obesity is associated with macrophage accumulation in adipose tissue. J. Clin. Investig..

[140] Harman-Boehm I., Blüher M., Redel H., Sion-Vardy N., Ovadia S., Avinoach E., Shai I., Klöting N., Stumvoll M., Bashan N. (2007). Macrophage infiltration into omental versus subcutaneous fat across different populations: Effect of regional adiposity and the comorbidities of obesity. J. Clin. Endocrinol. Metab..

[141] Moroni F., Ammirati E., Norata G.D., Magnoni M., Camici P.G. (2019). The Role of Monocytes and Macrophages in Human Atherosclerosis, Plaque Neoangiogenesis, and Atherothrombosis. Mediat. Inflamm..

[142] Loguinova M., Pinegina N., Kogan V., Vagida M., Arakelyan A., Shpektor A., Margolis L., Vasilieva E. (2018). Monocytes of Different Subsets in Complexes with Platelets in Patients with Myocardial Infarction. Thromb. Haemost..

[143] Singer K., DelProposto J., Morris D.L., Zamarron B., Mergian T., Maley N., Cho K.W., Geletka L., Subbaiah P., Muir L. (2014). Diet-induced obesity promotes myelopoiesis in hematopoietic stem cells. Mol. Metab..

[144] Nagareddy P.R., Kraakman M., Masters S.L., Stirzaker R.A., Gorman D.J., Grant R.W., Dragoljevic D., Hong E.S., Abdel-Latif A., Smyth S.S. (2014). Adipose tissue macrophages promote myelopoiesis and monocytosis in obesity. Cell Metab..

[145] Leite F., Leite Â., Santos A., Lima M., Barbosa J., Cosentino M., Ribeiro L. (2017). Predictors of Subclinical Inflammatory Obesity: Plasma Levels of Leptin, Very Low-Density Lipoprotein Cholesterol and CD14 Expression of CD16+ Monocytes. Obes. Facts.

[146] van der Valk E.S., Mulder D.S., Kouwenhoven T., Nagtzaam N.M.A., van Rossum E.F.C., Dik W.A., Leenen P.J.M. (2022). Monocyte adaptations in patients with obesity during a 1.5 year lifestyle intervention. Front. Immunol..

[147] Rogacev K.S., Ulrich C., Blömer L., Hornof F., Oster K., Ziegelin M., Cremers B., Grenner Y., Geisel J., Schlitt A. (2010). Monocyte heterogeneity in obesity subclinical atherosclerosis. Eur. Heart J..

[148] Powell L.A., Crowe P., Kankara C., McPeake J., McCance D.R., Young I.S., Trimble E.R., McGinty A. (2012). Restoration of adipose function in obese glucose-tolerant men following pioglitazone treatment is associated with CCAAT enhancer-binding protein β up-regulation. Clin. Sci..

[149] Pecht T., Haim Y., Bashan N., Shapiro H., Harman-Boehm I., Kirshtein B., Clément K., Shai I., Rudich A. (2016). Circulating Blood Monocyte Subclasses and Lipid-Laden Adipose Tissue Macrophages in Human Obesity. PLoS ONE.

[150] Kaur H., Adams-Huet B., Smith G., Jialal I. (2013). Increased neutrophil count in nascent metabolic syndrome. Metab. Syndr. Relat. Disord..

[151] van de Vyver M. (2023). Immunology of chronic low-grade inflammation: Relationship with metabolic function. J. Endocrinol..

[152] Trellakis S., Rydleuskaya A., Fischer C., Canbay A., Tagay S., Scherag A., Bruderek K., Schuler P.J., Brandau S. (2012). Low adiponectin, high levels of apoptosis and increased peripheral blood neutrophil activity in healthy obese subjects. Obes. Facts.

[153] Lee J.J., Jacobsen E.A., McGarry M.P., Schleimer R.P., Lee N.A. (2010). Eosinophils in health and disease: The LIAR hypothesis. Clin. Exp. Allergy.

[154] Calco G.N., Fryer A.D., Nie Z. (2020). Unraveling the connection between eosinophils and obesity. J. Leukoc. Biol..

[155] Cottam D.R., Schaefer P.A., Shaftan G.W., Velcu L., Angus L.D. (2002). Effect of surgically-induced weight loss on leukocyte indicators of chronic inflammation in morbid obesity. Obes. Surg..

[156] Johannsen N.M., Priest E.L., Dixit V.D., Earnest C.P., Blair S.N., Church T.S. (2010). Association of white blood cell subfraction concentration with fitness and fatness. Br. J. Sports Med..

[157] Sunadome H., Matsumoto H., Izuhara Y., Nagasaki T., Kanemitsu Y., Ishiyama Y., Morimoto C., Oguma T., Ito I., Murase K. (2020). Correlation between eosinophil count, its genetic background and body mass index: The Nagahama Study. Allergol. Int..

[158] Valentine Y., Nikolajczyk B.S. (2024). T cells in obesity-associated inflammation: The devil is in the details. Immunol. Rev..

[159] Liu R., Pugh G.H., Tevonian E., Thompson K., Lauffenburger D.A., Kern P.A., Nikolajczyk B.S. (2022). Regulatory T Cells Control Effector T Cell Inflammation in Human. Prediabetes. Diabetes.

[160] van der Weerd K., Dik W.A., Schrijver B., Schweitzer D.H., Langerak A.W., Drexhage H.A., Kiewiet R.M., van Aken M.O., van Huisstede A., van Dongen J.J. (2012). Morbidly obese human subjects have increased peripheral blood CD4+ T cells with skewing toward a Treg- and Th2-dominated phenotype. Diabetes.

[161] Ilavská S., Horváthová M., Szabová M., Nemessányi T., Jahnová E., Tulinská J., Líšková A., Wsolová L., Staruchová M., Volkovová K. (2012). Association between the human immune response and body mass index. Hum. Immunol..

[162] Lolmède K., Duffaut C., Zakaroff-Girard A., Bouloumié A. (2011). Immune cells in adipose tissue: Key players in metabolic disorders. Diabetes Metab..

[163] Martinez F.O., Gordon S. (2014). The M1 and M2 paradigm of macrophage activation: Time for reassessment. F1000Prime Rep..

[164] Berger A. (2000). Th1 and Th2 responses: What are they?. BMJ.

[165] Huang N., Dong H., Luo Y., Shao B. (2021). Th17 Cells in Periodontitis and Its Regulation by A20. Front. Immunol..

[166] Cinkajzlová A., Mráz M., Haluzík M. (2021). Adipose tissue immune cells in obesity, type 2 diabetes mellitus and cardiovascular diseases. J. Endocrinol..

[167] Méndez-García L.A., Solleiro-Villavicencio H., Bueno-Hernández N., Cérbulo-Vázquez A., Escobedo G., Esquivel-Velázquez M., Fonseca-Sánchez M.A. (2025). Role of the Th2-like Immune Response in Obesity: IL-4 as a Metabolic Regulator and IL-13 as an Effector of Muscle Energy Metabolism. Biomedicines.

[168] Nga H.T., Nguyen T.L., Yi H.S. (2024). T-Cell Senescence in Human Metabolic Diseases. Diabetes Metab. J..

[169] Sbierski-Kind J., Goldeck D., Buchmann N., Spranger J., Volk H.-D., Steinhagen-Thiessen E., Pawelec G., Demuth I., Spira D. (2020). T cell phenotypes associated with insulin resistance: Results from the Berlin Aging Study II. Immun. Ageing.

[170] Lau E.Y.M., Carroll E.C., A Callender L., A Hood G., Berryman V., Pattrick M., Finer S., Hitman G.A., Ackland G.L., Henson S.M. (2019). Type 2 diabetes is associated with the accumulation of senescent T cells. Clin. Exp. Immunol..

[171] Callender L.A., Carroll E.C., Garrod-Ketchley C., Schroth J., Bystrom J., Berryman V., Pattrick M., Campbell-Richards D., Hood G.A., Hitman G.A. (2021). Altered nutrient uptake causes mitochondrial dysfunction in senescent CD8+ EMRA T cells during type 2 diabetes. Front. Aging.

[172] Agabiti-Rosei C., Trapletti V., Piantoni S., Airò P., Tincani A., De Ciuceis C., Rossini C., Mittempergher F., Titi A., Portolani N. (2018). Decreased circulating T regulatory lymphocytes in obese patients undergoing bariatric surgery. PLoS ONE.

[173] Shevyrev D., Tereshchenko V. (2020). Treg Heterogeneity, Function, and Homeostasis. Front. Immunol..

[174] McClymont S.A., Putnam A.L., Lee M.R., Esensten J.H., Liu W., Hulme M.A., Hoffmüller U., Baron U., Olek S., Bluestone J.A. (2011). Plasticity of human regulatory T cells in healthy subjects and patients with type 1 diabetes. J. Immunol..

[175] Kleinewietfeld M., Hafler D.A. (2013). The plasticity of human Treg and Th17 cells and its role in autoimmunity. Semin. Immunol..

[176] Zhang S., Gang X., Yang S., Cui M., Sun L., Li Z., Wang G. (2021). The Alterations in and the Role of the Th17/Treg Balance in Metabolic Diseases. Front. Immunol..

[177] Shi L.Z., Wang R., Huang G., Vogel P., Neale G., Green D.R., Chi H. (2011). HIF1α-dependent glycolytic pathway orchestrates a metabolic checkpoint for the differentiation of TH17 and Treg cells. J. Exp. Med..

[178] Duan W., Ding Y., Yu X., Ma D., Yang B., Li Y., Huang L., Chen Z., Zheng J., Yang C. (2019). Metformin mitigates autoimmune insulitis by inhibiting Th1 and Th17 responses while promoting Treg production. Am. J. Transl. Res..

[179] Yang D.H., Lee H., Lee N., Shin M.S., Kang I., Kang K.S. (2021). Effector Memory CD8^+^ and CD4^+^ T Cell Immunity Associated with Metabolic Syndrome in Obese Children. Pediatr. Gastroenterol. Hepatol. Nutr..

[180] DeFuria J., Belkina A.C., Jagannathan-Bogdan M., Snyder-Cappione J., Carr J.D., Nersesova Y.R., Markham D., Strissel K.J., Watkins A.A., Zhu M. (2013). B cells promote inflammation in obesity and type 2 diabetes through regulation of T-cell function and an inflammatory cytokine profile. Proc. Natl. Acad. Sci. USA.

[181] Winer D.A., Winer S., Shen L., Wadia P.P., Yantha J., Paltser G., Tsui H., Wu P., Davidson M.G., Alonso M.N. (2011). B cells promote insulin resistance through modulation of T cells and production of pathogenic IgG antibodies. Nat. Med..

[182] Shen D.T., Qie Z.H., Zhao L.J., Pan L.J., Wang S.D., Liu C.X. (2025). Role of B lymphocyte ratio in development of type 2 diabetes mellitus: Results of a 7-year follow-up study. Front. Endocrinol..

[183] Frasca D., Diaz A., Romero M., Blomberg B.B. (2017). Human peripheral late/exhausted memory B cells express a senescent-associated secretory phenotype and preferentially utilize metabolic signaling pathways. Exp. Gerontol..

[184] Frasca D., Diaz A., Romero M., Thaller S., Blomberg B.B. (2019). Metabolic requirements of human pro-inflammatory B cells in aging and obesity. PLoS ONE.

[185] Frasca D., Romero M., Diaz A., Blomberg B.B. (2023). Obesity accelerates age defects in B cells, and weight loss improves B cell function. Immun. Ageing.

[186] Oleinika K., Slisere B., Catalán D., Rosser E.C. (2022). B cell contribution to immunometabolic dysfunction and impaired immune responses in obesity. Clin. Exp. Immunol..

[187] Agrawal S., Gollapudi S., Su H., Gupta S. (2011). Leptin activates human B cells to secrete TNF-α, IL-6, and IL-10 via JAK2/STAT3 and p38MAPK/ERK1/2 signaling pathway. J. Clin. Immunol..

[188] Bähr I., Spielmann J., Quandt D., Kielstein H. (2020). Obesity-Associated Alterations of Natural Killer Cells and Immunosurveillance of Cancer. Front. Immunol..

[189] Wensveen F.M., Jelenčić V., Valentić S., Šestan M., Wensveen T.T., Theurich S., Glasner A., Mendrila D., Štimac D., Wunderlich F.T. (2015). NK cells link obesity-induced adipose stress to inflammation and insulin resistance. Nat. Immunol..

[190] Theurich S., Tsaousidou E., Hanssen R., Lempradl A.M., Mauer J., Timper K., Schilbach K., Folz-Donahue K., Heilinger C., Sexl V. (2017). IL-6/Stat3-Dependent Induction of a Distinct, Obesity-Associated NK Cell Subpopulation Deteriorates Energy and Glucose Homeostasis. Cell Metab..

[191] Loguinova M., Sergeev N., Samsonova M., Sorokina A., Grebennikov D., Golodnikov I., Goncharenko A., Bocharov G., Laptev D., Khusainova R. (2025). Changes in circulating NK and innate-like T cells in type 1 and type 2 diabetes. Front. Immunol..

[192] Viel S., Besson L., Charrier E., Marçais A., Disse E., Bienvenu J., Walzer T., Dumontet C. (2017). Alteration of Natural Killer cell phenotype and function in obese individuals. Clin. Immunol..

[193] Kawai T., Autieri M.V., Scalia R. (2021). Adipose tissue inflammation and metabolic dysfunction in obesity. Am. J. Physiol. Cell Physiol..

[194] Esser N., L’homme L., De Roover A., Kohnen L., Scheen A.J., Moutschen M., Piette J., Legrand-Poels S., Paquot N. (2013). Obesity phenotype is related to NLRP3 inflammasome activity and immunological profile of visceral adipose tissue. Diabetologia.

[195] Furman D., Campisi J., Verdin E., Carrera-Bastos P., Targ S., Franceschi C., Ferrucci L., Gilroy D.W., Fasano A., Miller G.W. (2019). Chronic inflammation in the etiology of disease across the life span. Nat. Med..

[196] Talukdar S., Oh D.Y., Bandyopadhyay G., Li D., Xu J., McNelis J., Lu M., Li P., Yan Q., Zhu Y. (2012). Neutrophils mediate insulin resistance in mice fed a high-fat diet through secreted elastase. Nat. Med..

[197] Tynan G.A., Hearnden C.H., Oleszycka E., Lyons C.L., Coutts G., O’cOnnell J., Corrigan M.A., Lynch L., Campbell M., Callanan J.J. (2014). Endogenous oils derived from human adipocytes are potent adjuvants that promote IL-1α-dependent inflammation. Diabetes.

[198] Mansuy-Aubert V., Zhou Q.L., Xie X., Gong Z., Huang J.Y., Khan A.R., Aubert G., Candelaria K., Thomas S., Shin D.J. (2013). Imbalance between neutrophil elastase and its inhibitor α1-antitrypsin in obesity alters insulin sensitivity, inflammation, and energy expenditure. Cell Metab..

[199] Gupta A., Singh K., Fatima S., Ambreen S., Zimmermann S., Younis R., Krishnan S., Rana R., Gadi I., Schwab C. (2022). Neutrophil Extracellular Traps Promote NLRP3 Inflammasome Activation and Glomerular Endothelial Dysfunction in Diabetic Kidney Disease. Nutrients.

[200] García-Rubio J., León J., Redruello-Romero A., Pavón E., Cozar A., Tamayo F., Caba-Molina M., Salmerón J., Carazo Á. (2018). Cytometric analysis of adipose tissue reveals increments of adipocyte progenitor cells after weight loss induced by bariatric surgery. Sci. Rep..

[201] Weinstock A., Moura Silva H., Moore K.J., Schmidt A.M., Fisher E.A. (2020). Leukocyte Heterogeneity in Adipose Tissue, Including in Obesity. Circ. Res..

[202] Hu Y., Chakarov S. (2023). Eosinophils in obesity and obesity-associated disorders. Discov. Immunol..

[203] Knights A.J., Vohralik E.J., Houweling P.J., Stout E.S., Norton L.J., Alexopoulos S.J., Yik J.J., Jusoh H.M., Olzomer E.M., Bell-Anderson K.S. (2020). Eosinophil function in adipose tissue is regulated by Krüppel-like factor 3 (KLF3). Nat. Commun..

[204] Qiu Y., Nguyen K.D., Odegaard J.I., Cui X., Tian X., Locksley R.M., Palmiter R.D., Chawla A. (2014). Eosinophils and type 2 cytokine signaling in macrophages orchestrate development of functional beige fat. Cell.

[205] Wu D., Molofsky A.B., Liang H.-E., Ricardo-Gonzalez R.R., Jouihan H.A., Bando J.K., Chawla A., Locksley R.M. (2011). Eosinophils sustain adipose alternatively activated macrophages associated with glucose homeostasis. Science.

[206] Lee E.-H., Itan M., Jang J., Gu H.-J., Rozenberg P., Mingler M.K., Wen T., Yoon J., Park S.-Y., Roh J.Y. (2018). Eosinophils support adipocyte maturation and promote glucose tolerance in obesity. Sci. Rep..

[207] Fabbiano S., Suárez-Zamorano N., Rigo D., Veyrat-Durebex C., Stevanovic Dokic A., Colin D.J., Trajkovski M. (2016). Caloric Restriction Leads to Browning of White Adipose Tissue through Type 2 Immune Signaling. Cell Metab..

[208] Bolus W.R., Hasty A.H. (2019). Contributions of innate type 2 inflammation to adipose function. J. Lipid Res..

[209] Moussa K., Gurung P., Adams-Huet B., Devaraj S., Jialal I. (2019). Increased eosinophils in adipose tissue of metabolic syndrome. J. Diabetes Complicat..

[210] Poglio S., De Toni-Costes F., Arnaud E., Laharrague P., Espinosa E., Casteilla L., Cousin B. (2010). Adipose tissue as a dedicated reservoir of functional mast cell progenitors. Stem Cells.

[211] Lopez-Perez D., Redruello-Romero A., Garcia-Rubio J., Arana C., Garcia-Escudero L.A., Tamayo F., Puentes-Pardo J.D., Moreno-SanJuan S., Salmeron J., Blanco A. (2021). In Patients With Obesity, the Number of Adipose Tissue Mast Cells Is Significantly Lower in Subjects With Type 2 Diabetes. Front. Immunol..

[212] Gurung P., Moussa K., Adams-Huet B., Devaraj S., Jialal I. (2019). Increased mast cell abundance in adipose tissue of metabolic syndrome: Relevance to the proinflammatory state and increased adipose tissue fibrosis. Am. J. Physiol. Endocrinol. Metab..

[213] Divoux A., Moutel S., Poitou C., Lacasa D., Veyrie N., Aissat A., Arock M., Guerre-Millo M., Clément K. (2012). Mast cells in human adipose tissue: Link with morbid obesity, inflammatory status, and diabetes. J. Clin. Endocrinol. Metab..

[214] Suzukawa M., Nagase H., Ogahara I., Han K., Tashimo H., Shibui A., Koketsu R., Nakae S., Yamaguchi M., Ohta K. (2011). Leptin enhances survival and induces migration, degranulation, and cytokine synthesis of human basophils. J. Immunol..

[215] Shi M.A., Shi G.P. (2012). Different roles of mast cells in obesity and diabetes: Lessons from experimental animals and humans. Front. Immunol..

[216] Salamon P., Shoham N.G., Puxeddu I., Paitan Y., Levi-Schaffer F., Mekori Y.A. (2008). Human mast cells release oncostatin M on contact with activated T cells: Possible biologic relevance. J. Allergy Clin. Immunol..

[217] Apostolopoulos V., de Courten M.P., Stojanovska L., Blatch G.L., Tangalakis K., de Courten B. (2016). The complex immunological and inflammatory network of adipose tissue in obesity. Mol. Nutr. Food Res..

[218] Krystel-Whittemore M., Dileepan K.N., Wood J.G. (2016). Mast Cell: A Multi-Functional Master Cell. Front. Immunol..

[219] Yunna C., Mengru H., Lei W., Weidong C. (2020). Macrophage M1/M2 polarization. Eur. J. Pharmacol..

[220] Luo M., Zhao F., Cheng H., Su M., Wang Y. (2024). Macrophage polarization: An important role in inflammatory diseases. Front. Immunol..

[221] Santamarina A.B., Mennitti L.V., de Souza E.A., Mesquita L.M.S., Noronha I.H., Vasconcelos J.R.C., Prado C.M., Pisani L.P. (2023). A low-carbohydrate diet with different fatty acids’ sources in the treatment of obesity: Impact on insulin resistance and adipogenesis. Clin. Nutr..

[222] Brandt S.L., Serezani C.H. (2017). Too much of a good thing: How modulating LTB4 actions restore host defense in homeostasis or disease. Semin. Immunol..

[223] Li X., Ren Y., Chang K., Wu W., Griffiths H.R., Lu S., Gao D. (2023). Adipose tissue macrophages as potential targets for obesity and metabolic diseases. Front. Immunol..

[224] Kratz M., Coats B.R., Hisert K.B., Hagman D., Mutskov V., Peris E., Schoenfelt K.Q., Kuzma J.N., Larson I., Billing P.S. (2014). Metabolic dysfunction drives a mechanistically distinct proinflammatory phenotype in adipose tissue macrophages. Cell Metab..

[225] Bourlier V., Zakaroff-Girard A., Miranville A., De Barros S., Maumus M., Sengenes C., Galitzky J., Lafontan M., Karpe F., Frayn K. (2008). Remodeling phenotype of human subcutaneous adipose tissue macrophages. Circulation.

[226] Hill D.A., Lim H.-W., Kim Y.H., Ho W.Y., Foong Y.H., Nelson V.L., Nguyen H.C.B., Chegireddy K., Kim J., Habertheuer A. (2018). Distinct macrophage populations direct inflammatory versus physiological changes in adipose tissue. Proc. Natl. Acad. Sci. USA.

[227] Xu R., Vujić N., Bianco V., Reinisch I., Kratky D., Krstic J., Prokesch A. (2024). Lipid-associated macrophages between aggravation and alleviation of metabolic diseases. Trends Endocrinol. Metab..

[228] Jaitin D.A., Adlung L., Thaiss C.A., Weiner A., Li B., Descamps H., Lundgren P., Bleriot C., Liu Z., Deczkowska A. (2019). Lipid-Associated Macrophages Control Metabolic Homeostasis in a Trem2-Dependent Manner. Cell.

[229] Fang X.H., Li Z.J., Liu C.Y., Mor G., Liao A.H. (2024). Macrophage memory: Types, mechanisms, and its role in health and disease. Immunology.

[230] Netea M.G., Domínguez-Andrés J., Barreiro L.B., Chavakis T., Divangahi M., Fuchs E., Joosten L.A.B., van der Meer J.W.M., Mhlanga M.M., Mulder W.J.M. (2020). Defining trained immunity and its role in health and disease. Nat. Rev. Immunol..

[231] Caslin H.L., Cottam M.A., Piñon J.M., Boney L.Y., Hasty A.H. (2023). Weight cycling induces innate immune memory in adipose tissue macrophages. Front. Immunol..

[232] Chen S., Yang J., Wei Y., Wei X. (2020). Epigenetic regulation of macrophages: From homeostasis maintenance to host defense. Cell. Mol. Immunol..

[233] Russo S., Kwiatkowski M., Govorukhina N., Bischoff R., Melgert B.N. (2021). Meta-Inflammation and Metabolic Reprogramming of Macrophages in Diabetes and Obesity: The Importance of Metabolites. Front. Immunol..

[234] Johnson C., Iv C.D., Shan H., Shao Y., Sun Y., Lu Y., Saaoud F., Xu K., Nanayakkara G., Fang P. (2021). A Novel Subset of CD95+ Pro-Inflammatory Macrophages Overcome miR155 Deficiency and May Serve as a Switch From Metabolically Healthy Obesity to Metabolically Unhealthy Obesity. Front. Immunol..

[235] Ruggiero A.D., Vemuri R., Block M., DeStephanis D., Davis M., Chou J., Williams A., Brock A., Das S.K., Kavanagh K. (2022). Macrophage Phenotypes and Gene Expression Patterns Are Unique in Naturally Occurring Metabolically Healthy Obesity. Int. J. Mol. Sci..

[236] Kintscher U., Hartge M., Hess K., Foryst-Ludwig A., Clemenz M., Wabitsch M., Fischer-Posovszky P., Barth T.F., Dragun D., Skurk T. (2008). T-lymphocyte infiltration in visceral adipose tissue: A primary event in adipose tissue inflammation and the development of obesity-mediated insulin resistance. Arterioscler. Thromb. Vasc. Biol..

[237] Papatriantafyllou M. (2013). CD4+ T cell activation by adipocytes in obesity. Nat. Rev. Immunol..

[238] Song J., Deng T. (2020). The Adipocyte and Adaptive Immunity. Front Immunol..

[239] McLaughlin T., Liu L.-F., Lamendola C., Shen L., Morton J., Rivas H., Winer D., Tolentino L., Choi O., Zhang H. (2014). T-cell profile in adipose tissue is associated with insulin resistance and systemic inflammation in humans. Arterioscler. Thromb. Vasc. Biol..

[240] Chung Y., Chang J.Y., Soedono S., Julietta V., Joo E.J., Kwon S.H., Choi S.I., Kim Y.J., Cho K.W. (2025). Distinct T Cell Subset Profiles and T-Cell Receptor Signatures in Metabolically Unhealthy Obesity. Int. J. Mol. Sci..

[241] Shirakawa K., Sano M. (2021). T Cell Immunosenescence in Aging, Obesity, and Cardiovascular Disease. Cells.

[242] Shirakawa K., Sano M. (2023). Drastic transformation of visceral adipose tissue and peripheral CD4 T cells in obesity. Front. Immunol..

[243] Zou J., Lai B., Zheng M., Chen Q., Jiang S., Song A., Huang Z., Shi P., Tu X., Wang D. (2018). CD4+ T cells memorize obesity and promote weight regain. Cell Mol. Immunol..

[244] Shirakawa K., Endo J., Katsumata Y., Yamamoto T., Kataoka M., Isobe S., Yoshida N., Fukuda K., Sano M. (2017). Negative legacy of obesity. PLoS ONE.

[245] Kolodin D., van Panhuys N., Li C., Magnuson A.M., Cipolletta D., Miller C.M., Wagers A., Germain R.N., Benoist C., Mathis D. (2015). Antigen- and cytokine-driven accumulation of regulatory T cells in visceral adipose tissue of lean mice. Cell Metab..

[246] Croce S., Avanzini M.A., Regalbuto C., Cordaro E., Vinci F., Zuccotti G., Calcaterra V. (2021). Adipose tissue immunomodulation and treg/th17 imbalance in the impaired glucose metabolism of children with obesity. Children.

[247] Zeng Q., Sun X., Xiao L., Xie Z., Bettini M., Deng T. (2018). A Unique Population: Adipose-Resident Regulatory T Cells. Front. Immunol..

[248] Pereira S., Teixeira L., Aguilar E., Oliveira M., Savassi-Rocha A., Pelaez J.N., Capettini L., Diniz M.T., Ferreira A., Alvarez-Leite J. (2014). Modulation of adipose tissue inflammation by FOXP3+ Treg cells, IL-10, and TGF-β in metabolically healthy class III obese individuals. Nutrition.

[249] Jiang E., Perrard X.D., Yang D., Khan I.M., Perrard J.L., Smith C.W., Ballantyne C.M., Wu H. (2014). Essential role of CD11a in CD8+ T-cell accumulation and activation in adipose tissue. Arterioscler. Thromb. Vasc. Biol..

[250] Chen X., Wang S., Huang Y., Zhao X., Jia X., Meng G., Zheng Q., Zhang M., Wu Y., Wang L. (2020). Obesity Reshapes Visceral Fat-Derived MHC I Associated-Immunopeptidomes and Generates Antigenic Peptides to Drive CD8+ T Cell Responses. iScience.

[251] Wang L., Sun P., Wu Y., Wang L. (2021). Metabolic tissue-resident CD8+ T cells: A key player in obesity-related diseases. Obes. Rev..

[252] Turbitt W.J., Buchta Rosean C., Weber K.S., Norian L.A. (2020). Obesity and CD8 T cell metabolism: Implications for anti-tumor immunity and cancer immunotherapy outcomes. Immunol. Rev..

[253] Harmon D.B., Srikakulapu P., Kaplan J.L., Oldham S.N., McSkimming C., Garmey J.C., Perry H.M., Kirby J.L., Prohaska T.A., Gonen A. (2016). Protective Role for B-1b B Cells and IgM in Obesity-Associated Inflammation, Glucose Intolerance, and Insulin Resistance. Arterioscler. Thromb. Vasc. Biol..

[254] Ying W., Wollam J., Ofrecio J.M., Bandyopadhyay G., El Ouarrat D., Lee Y.S., Oh D.Y., Li P., Osborn O., Olefsky J.M. (2017). Adipose tissue B2 cells promote insulin resistance through leukotriene LTB4/LTB4R1 signaling. J. Clin. Investig..

[255] Winer D.A., Winer S., Shen L., Chng M.H., Engleman E.G. (2012). B lymphocytes as emerging mediators of insulin resistance. Int. J. Obes. Suppl..

[256] Myles A., Sanz I., Cancro M.P. (2019). T-bet+ B cells: A common denominator in protective and autoreactive antibody responses?. Curr. Opin. Immunol..

[257] Hägglöf T., Vanz C., Kumagai A., Dudley E., Ortega V., Siller M., Parthasarathy R., Keegan J., Koenigs A., Shute T. (2022). T-bet+ B cells accumulate in adipose tissue and exacerbate metabolic disorder during obesity. Cell Metab..

[258] Fernø J., Strand K., Mellgren G., Stiglund N., Björkström N.K. (2020). Natural Killer Cells as Sensors of Adipose Tissue Stress. Trends Endocrinol. Metab..

[259] Lee B.-C., Kim M.-S., Pae M., Yamamoto Y., Eberlé D., Shimada T., Kamei N., Park H.-S., Sasorith S., Woo J.R. (2016). Adipose Natural Killer Cells Regulate Adipose Tissue Macrophages to Promote Insulin Resistance in Obesity. Cell Metab..

[260] O’Sullivan T.E., Rapp M., Fan X., Weizman O.-E., Bhardwaj P., Adams N.M., Walzer T., Dannenberg A.J., Sun J.C. (2016). Adipose-Resident Group 1 Innate Lymphoid Cells Promote Obesity-Associated Insulin Resistance. Immunity.

[261] Aouadi M., Tencerova M., Vangala P., Yawe J.C., Nicoloro S.M., Amano S.U., Cohen J.L., Czech M.P. (2013). Gene silencing in adipose tissue macrophages regulates whole-body metabolism in obese mice. Proc. Natl. Acad. Sci. USA.

[262] Wang H., Shen L., Sun X., Liu F., Feng W., Jiang C., Chu X., Ye X., Jiang C., Wang Y. (2019). Adipose group 1 innate lymphoid cells promote adipose tissue fibrosis and diabetes in obesity. Nat. Commun..

[263] Molofsky A.B., Van Gool F., Liang H.-E., Van Dyken S.J., Nussbaum J.C., Lee J., Bluestone J.A., Locksley R.M. (2015). Interleukin-33 and Interferon-γ Counter-Regulate Group 2 Innate Lymphoid Cell Activation during Immune Perturbation. Immunity.

[264] Baragetti A., Da Dalt L., Moregola A., Svecla M., Terenghi O., Mattavelli E., De Gaetano L.N., Uboldi P., Catapano A.L., Norata G.D. (2023). Neutrophil aging exacerbates high fat diet induced metabolic alterations. Metabolism.

[265] Todosenko N., Khaziakhmatova O., Malashchenko V., Yurova K., Bograya M., Beletskaya M., Vulf M., Mikhailova L., Minchenko A., Soroko I. (2023). Adipocyte- and Monocyte-Mediated Vicious Circle of Inflammation and Obesity (Review of Cellular and Molecular Mechanisms). Int. J. Mol. Sci..

[266] Kiran S., Kumar V., Murphy E.A., Enos R.T., Singh U.P. (2021). High Fat Diet-Induced CD8+ T Cells in Adipose Tissue Mediate Macrophages to Sustain Low-Grade Chronic Inflammation. Front. Immunol..

[267] Winer S., Chan Y., Paltser G., Truong D., Tsui H., Bahrami J., Dorfman R., Wang Y., Zielenski J., Mastronardi F. (2009). Normalization of obesity-associated insulin resistance through immunotherapy. Nat. Med..

[268] Fabbrini E., Cella M., Mccartney S.A., Fuchs A., Abumrad N.A., Pietka T.A., Chen Z., Finck B.N., Han D.H., Magkos F. (2013). Association between specific adipose tissue CD4+ T-cell populations and insulin resistance in obese individuals. Gastroenterology.

[269] Bradley D., Smith A.J., Blaszczak A., Shantaram D., Bergin S.M., Jalilvand A., Wright V., Wyne K.L., Dewal R.S., Baer L.A. (2022). Interferon gamma mediates the reduction of adipose tissue regulatory T cells in human obesity. Nat. Commun..

[270] Dam A.D.v. (2017). INFLAMED FAT: Immune Modulation of Adipose Tissue and Lipid Metabolism. https://hdl.handle.net/1887/54937.

[271] Cornejo-Pareja I., Clemente-Postigo M., Tinahones F.J. (2019). Metabolic and Endocrine Consequences of Bariatric Surgery. Front. Endocrinol..

[272] Fischer L., Hildebrandt C., Bruckner T., Kenngott H., Linke G.R., Gehrig T., Büchler M.W., Müller-Stich B.P. (2012). Excessive weight loss after sleeve gastrectomy: A systematic review. Obes. Surg..

[273] O’brien P.E., Hindle A., Brennan L., Skinner S., Burton P., Smith A., Crosthwaite G., Brown W. (2018). Long-Term Outcomes After Bariatric Surgery: A Systematic Review and Meta-analysis of Weight Loss at 10 or More Years for All Bariatric Procedures and a Single-Centre Review of 20-Year Outcomes After Adjustable Gastric Banding. Obes. Surg..

[274] Meyer-Gerspach A.C., Peterli R., Moor M., Madörin P., Schötzau A., Nabers D., Borgwardt S., Beglinger C., Bieri O., Wölnerhanssen B.K. (2019). Quantification of Liver, Subcutaneous, and Visceral Adipose Tissues by MRI Before and After Bariatric Surgery. Obes. Surg..

[275] Hansen M., Lund M.T., Gregers E., Kraunsøe R., Van Hall G., Helge J.W., Dela F. (2015). Adipose tissue mitochondrial respiration lipolysis before after a weight loss by diet and RYGB. Obesity.

[276] Torriani M., Oliveira A.L., Azevedo D.C., Bredella M.A., Yu E.W. (2015). Effects of Roux-en-Y gastric bypass surgery on visceral and subcutaneous fat density by computed tomography. Obes. Surg..

[277] Osorio-Conles Ó., Vidal J., de Hollanda A. (2021). Impact of Bariatric Surgery on Adipose Tissue Biology. J. Clin. Med..

[278] Andersson D.P., Hogling D.E., Thorell A., Toft E., Qvisth V., Näslund E., Thörne A., Wirén M., Löfgren P., Hoffstedt J. (2014). Changes in subcutaneous fat cell volume and insulin sensitivity after weight loss. Diabetes Care.

[279] Labrecque J., Laforest S., Michaud A., Biertho L., Tchernof A. (2017). Impact of Bariatric Surgery on White Adipose Tissue Inflammation. Can. J. Diabetes.

[280] Hoffstedt J., Andersson D.P., Hogling D.E., Theorell J., Näslund E., Thorell A., Ehrlund A., Rydén M., Arner P. (2017). Long-term Protective Changes in Adipose Tissue After Gastric Bypass. Diabetes Care.

[281] Rizk N.M., Fadel A., AlShammari W., Younes N., Bashah M. (2021). The Immunophenotyping Changes of Peripheral CD4+ T Lymphocytes and Inflammatory Markers of Class III Obesity Subjects After Laparoscopic Gastric Sleeve Surgery-A Follow-Up Study. J. Inflamm. Res..

[282] Khosravi-Largani M., Nojomi M., Aghili R., Otaghvar H.A., Tanha K., Seyedi S.H.S., Mottaghi A. (2019). Evaluation of all Types of Metabolic Bariatric Surgery and its Consequences: A Systematic Review and Meta-Analysis. Obes. Surg..

[283] Arica P.C., Aydin S., Zengin U., Kocael A., Orhan A., Zengin K., Gelisgen R., Taskin M., Uzun H. (2018). The Effects on Obesity Related Peptides of Laparoscopic Gastric Band Applications in Morbidly Obese Patients. J. Investig. Surg..

[284] Šebunova N., Štšepetova J., Kullisaar T., Suija K., Rätsep A., Junkin I., Soeorg H., Lember M., Sillakivi T., Mändar R. (2022). Changes in adipokine levels and metabolic profiles following bariatric surgery. BMC Endocr. Disord..

[285] Lapointe M., Poirier P., Martin J., Bastien M., Auclair A., Cianflone K. (2014). Omentin changes following bariatric surgery and predictive links with biomarkers for risk of cardiovascular disease. Cardiovasc. Diabetol..

[286] Dai X., Zhao W., Zhan J., Zeng S., Ran D., Zhang H., Song Z., Song K.H., Wu L. (2017). B cells present skewed profile and lose the function of supporting T cell inflammation after Roux-en-Y gastric bypass. Int. Immunopharmacol..

[287] Katsogiannos P., Kamble P.G., Pereira M.J., Sundbom M., Carlsson P., Eriksson J.W., Espes D. (2021). Changes in Circulating Cytokines and Adipokines After RYGB in Patients with and without Type 2 Diabetes. Obesity.

[288] Bratti L.O.S., do Carmo Í.A.R., Vilela T.F., Souza L.C., Moraes A.C.R., Filippin-Monteiro F.B. (2021). Bariatric surgery improves clinical outcomes and adiposity biomarkers but not inflammatory cytokines SAA and MCP-1 after a six-month follow-up. Scand. J. Clin. Lab. Investig..

[289] Palomäki V.A., Lehenkari P., Meriläinen S., Karttunen T.J., Koivukangas V. (2023). Dynamics of adipose tissue macrophage populations after gastric bypass surgery. Obesity.

[290] Aron-Wisnewsky J., Tordjman J., Poitou C., Darakhshan F., Hugol D., Basdevant A., Aissat A., Guerre-Millo M., ClémEnt K. (2009). Human adipose tissue macrophages: M1 and m2 cell surface markers in subcutaneous and omental depots and after weight loss. J. Clin. Endocrinol. Metab..

[291] Hagman D.K., Larson I., Kuzma J.N., Cromer G., Makar K., Rubinow K.B., Foster-Schubert K.E., van Yserloo B., Billing P.S., Landerholm R.W. (2017). The short-term and long-term effects of bariatric/metabolic surgery on subcutaneous adipose tissue inflammation in humans. Metabolism.

[292] Kristensen M.D., Lund M.T., Hansen M., Poulsen S.S., Ploug T., Dela F., Helge J.W., Prats C. (2017). Macrophage Area Content and Phenotype in Hepatic and Adipose Tissue in Patients with Obesity Undergoing Roux-en-Y Gastric Bypass. Obesity.

[293] Lecoutre S., Rebière C., Marcelin G., Clément K. (2024). How does bariatric surgery remodel adipose tissue?. Ann. Endocrinol..

[294] Zamarron B.F., Mergian T.A., Cho K.W., Martinez-Santibanez G., Luan D., Singer K., DelProposto J.L., Geletka L.M., Muir L.A., Lumeng C.N. (2017). Macrophage Proliferation Sustains Adipose Tissue Inflammation in Formerly Obese Mice. Diabetes.

[295] Schmitz J., Evers N., Awazawa M., Nicholls H., Brönneke H., Dietrich A., Mauer J., Blüher M., Brüning J. (2016). Obesogenic memory can confer long-term increases in adipose tissue but not liver inflammation and insulin resistance after weight loss. Mol. Metab..

[296] Cottam M.A., Caslin H.L., Winn N.C., Hasty A.H. (2022). Multiomics reveals persistence of obesity-associated immune cell phenotypes in adipose tissue during weight loss and weight regain in mice. Nat. Commun..

[297] van Baak M.A., Mariman E.C.M. (2023). Obesity-induced and weight-loss-induced physiological factors affecting weight regain. Nat. Rev. Endocrinol..

[298] van Baak M.A., Mariman E.C.M. (2019). Mechanisms of weight regain after weight loss-the role of adipose tissue. Nat. Rev. Endocrinol..

[299] Cottam M.A., Itani H.A., Beasley A.A., Hasty A.H. (2018). Links between Immunologic Memory and Metabolic Cycling. J. Immunol..

[300] Song W.M., Colonna M. (2018). Immune Training Unlocks Innate Potential. Cell.

[301] Giovenzana A., Bezzecchi E., Bichisecchi A., Cardellini S., Ragogna F., Pedica F., Invernizzi F., Di Filippo L., Tomajer V., Aleotti F. (2024). Fat-to-blood recirculation of partially dysfunctional PD-1+CD4 Tconv cells is associated with dysglycemia in human obesity. iScience.

[302] Hinte L.C., Castellano-Castillo D., Ghosh A., Melrose K., Gasser E., Noé F., Massier L., Dong H., Sun W., Hoffmann A. (2024). Adipose Tissue Retains an Epigenetic Memory of Obesity after Weight Loss. Nature.

[303] Sams V.G., Blackledge C., Wijayatunga N., Barlow P., Mancini M., Mancini G., Moustaid-Moussa N. (2016). Effect of bariatric surgery on systemic and adipose tissue inflammation. Surg. Endosc..

[304] Liu Z., Yu S., Jin X., Sheng L., YanMu M.R., Gao J., Lu J., Lei T. (2025). The Clinical Application of GLP-1RAs and GLP-1/GIP Dual Receptor Agonists Based on Pharmacological Mechanisms: A Review. Drug Des. Devel. Ther..

[305] Zheng Z., Zong Y., Ma Y., Tian Y., Pang Y., Zhang C., Gao J. (2024). Glucagon-like peptide-1 receptor: Mechanisms and advances in therapy. Signal Transduct. Target Ther..

[306] Alharbi S.H. (2024). Anti-inflammatory role of glucagon-like peptide 1 receptor agonists and its clinical implications. Ther. Adv. Endocrinol. Metab..

[307] Yaribeygi H., Maleki M., Jamialahmadi T., Sahebkar A. (2024). Anti-inflammatory benefits of semaglutide: State of the art. J. Clin. Transl. Endocrinol..

[308] Comşa A.D., Comşa H., Cismaru G., Roşu R., Pop D. (2026). Anti-inflammatory Pathways of Novel Anti-diabetic Therapies. A Literature Review. Vivo.

[309] Wang X., Qi M., Yang L., Yang L., Wang X., Zhang F., Cui Y., Wang D., Wang Y., Lv W. (2025). GLP-1 receptor agonists synergistic effects of metabolic reprogramming and cardioprotection. Front. Endocrinol..

[310] Chamchoum E., Katrib N., Nassif N., Ratel Y., Rida M.A. (2025). Glucagon-like peptide and its receptor agonists for the treatment of rheumatic diseases. World J. Exp. Med..

