# Supplementary material for: From Metabolically Healthy to Unhealthy Obesity Through Low-Grade Inflammation
Source: Biomedicines. 2026 May 20;14(5):1161. doi: 10.3390/biomedicines14051161 (PMC13204051; doi:10.3390/biomedicines14051161)
Supplement: Supplementary file 1 [file biomedicines-14-01161-s001.zip › biomedicines-4221320-supplementary.pdf]

Table S1. Anthropometric and biochemical characteristics of healthy individuals and in MHO and MUO.

Parameters	Healthy Individuals	MHO	MUO	References
Body weight, kg±SD	70.9 ± 12.1	95.7 ± 14.6	97.2 ± 15.0, p≤0.001 for all	[12]
BMI, kg/m²±SD	25.4 ± 2.8	34.9 ± 4.4	35.1 ± 4.7, p≤0.001 for all	[12]
WHR, m ±SD	0.86 ± 0.04	0.91 ± 0.04	0.94 ± 0.04, p≤0.001 for healthy and MHO vs MUO	[31]
Wrist circumference, cm±SD	87 ± 9	107 ± 9	111 ± 9 (p≤0.001 for all)	[48]
Fat content, %±SD	15.91 ± 4.02	23.27 ± 4.94	24.44 ± 6.04, p≤0.001 for healthy vs MUO	[31]
Visceral fat area, cm²±SD	71.42 ± 23.6	107.46 ± 30.78	109.77 ± 33.76, p≤0.001 for healthy and MHO vs MUO	[31]
SBP, mmHg±SD	129.8 ± 18.6	132.1 ± 17.3	138.6 ± 16.9, p≤0.001 for all	[12]
DBP, mmHg±SD	73.9 ± 10.2	74.9 ± 10.4	77.0 ± 10.5, p≤0.001 for all	[12]
Fasting glucose, mg/dL±SD	90.8 ± 26.2	87.3 ± 8.6	107.8 ± 38.0, p≤0.001 for all	[12]
HbA1c, %±SD	5.5 ± 0.4	5.7 ± 0.4	6.2 ± 0.9, p≤0.0001 for MUO vs healthy	[49]
HOMA-IR±SD	2.81 ± 1.55	3.83 ± 1.93	5.68 ± 3.05 (p≤0.001 for all)	[48]
Cholesterol, mmol/L±SD	4.20 ± 1.06	4.31 ± 1.04	5.13 ± 1.17, p≤0.001	[50]
HDL, mg/dL±SD	53.2 ± 15.5	55.7 ± 14.0	44.3 ± 10.8, p≤0.001 for all	[12]
LDL, mmol/L±SD	1.85 ± 0.66	2.01 ± 0.84	2.37 ± 0.73, p≤0.001	[50]
Triglycerides, mg/dL±SD	127.6 ± 83.7	150.5 ± 108.0	174.2 ± 88.1, p≤0.001 for all	[12]

Notes to Table S1: MHO- metabolically healthy obesity, MUO- metabolically unhealthy obesity, BMI- body mass index, WHR- waist-hip ratio, SBP- systolic blood pressure, DBP- diastolic blood pressure, HDL- high density lipoproteins, LDL- low density lipoproteins.
